# Complex Regulation by Apetala2 Domain-Containing Transcription Factors Revealed through Analysis of the Stress-Responsive *TdCor410b* Promoter from Durum Wheat

**DOI:** 10.1371/journal.pone.0058713

**Published:** 2013-03-19

**Authors:** Omid Eini, Nannan Yang, Tatiana Pyvovarenko, Katherine Pillman, Natalia Bazanova, Natalia Tikhomirov, Serik Eliby, Neil Shirley, Shoba Sivasankar, Scott Tingey, Peter Langridge, Maria Hrmova, Sergiy Lopato

**Affiliations:** 1 Australian Centre for Plant Functional Genomics, University of Adelaide, Glen Osmond, South Australia, Australia; 2 DuPont Ag Biotechnology, Johnston, Iowa, United States of America; 3 DuPont Ag Biotechnology, Wilmington, Delaware, United States of America; University of Massachusetts Amherst, United States of America

## Abstract

Expression of the wheat dehydrin gene *Cor410b* is induced several fold above its non-stressed levels upon exposure to stresses such as cold, drought and wounding. Deletion analysis of the *TdCor410b* promoter revealed a single functional C-repeat (CRT) element. Seven transcription factors (TFs) were shown to bind to this CRT element using yeast one-hybrid screens of wheat and barley cDNA libraries, of which only one belonged to the DREB class of TFs. The remaining six encoded ethylene response factors (ERFs) belong to three separate subfamilies. Analysis of binding selectivity of these TFs indicated that all seven could bind to the CRT element (GCCGAC), and that three of the six ERFs could bind both to the CRT element and the ethylene-responsive GCC-box (GCCGCC). The TaERF4 subfamily members specifically bound the CRT element, and did not bind either the GCC-box or DRE element (ACCGAC). Molecular modeling and site-directed mutagenesis identified a single residue Pro42 in the Apetala2 (AP2) domain of TaERF4-like proteins that is conserved in monocotyledonous plants and is responsible for the recognition selectivity of this subfamily. We suggest that both DREB and ERF proteins regulate expression of the *Cor410b* gene through a single, critical CRT element. Members of the TaERF4 subfamily are specific, positive regulators of *Cor410b* gene expression.

## Introduction

Among various transcription factors (TFs) reported to be associated with abiotic and biotic stress tolerance in plants, the most widely studied are the drought-responsive element (DRE) binding proteins (DREBs) and the ethylene response factors (ERFs). The DREB proteins, known also as the C-repeat (CRT) binding factors (CBFs), regulate expression of drought/cold stress-related genes by binding to the CRT element (GCCGAC) [1]–[6], while the ERFs are known to bind to the GCC box (GCCGCC) [7]–[13] of gene promoters. Both families of proteins contain the Apetala2 (AP2) domain, while the CBF/DREB proteins are distinguished further by the presence of two additional regions, PKKP/RAGRxKFxETRHP (abbreviated PKKPAGR) and DSAWR, which are located immediately upstream and downstream, respectively, of the AP2 DNA-binding domain [14]. Although the ERF proteins are generally known to bind only the GCC box, at least two ERFs, one from pepper and the other from wheat, have previously been shown to associate with both the GCC box and the CRT/DRE element [15], [16]. This dual binding has been suggested to be responsible for dual responses triggered by a single ERF under different environmental conditions.

Dehydrins, a class of Late Embryogenesis Abundant (LEA) proteins, constitute an important family of abiotic-stress-responsive genes [17]. These proteins are constitutively expressed in mature embryos and endosperm under normal growth conditions. Their expression is further activated several fold in other plant tissues, upon exposure to stresses with an osmotic component such as drought, high salinity and cold [18]. The promoters of genes encoding dehydrins are strongly activated in vegetative tissues by these stresses.

The *Cor410* gene was originally identified as a gene encoding a LEA protein that accumulates to similar levels in root, crown and leaf tissues of freezing-tolerant Gramineae during cold acclimation [19]. It has recently been demonstrated that levels of *TaCor410* transcripts are highest for low-temperature tolerant wheat genotypes and lowest for tender genotypes [20]. Highest transcript levels in crown and leaf tissues of cold-tolerant wheat (*Triticum aestivum* L.) were observed on the second day of cold acclimation [20]. Immunolocalisation of the TaCor410 protein revealed that it accumulated close to the plasma membrane of cells in the vascular transition area, where freezing-induced dehydration is likely to be more severe [21]. This finding suggested that the TaCor410 protein may function in protection of cell membranes under freezing and/or dehydration conditions. Constitutive expression of the *TaCor410* gene in transgenic strawberry at a level comparable to that in wheat after cold acclimation resulted in some improvement in freezing tolerance, although no improvement was detected in the absence of acclimation [22]. The authors suggested the need for other protein partners that could be induced during acclimation for activation of *TaCor410*. The closest homologues of *TaCor410* reported in other plant species are *AtCOR47* from *Arabidopsis* [23], *HvDhn8* from barley [24] and *OsDhn1* from rice [25]. The expression of *AtCOR47* and *HvDhn8* is strongly induced by cold, but also up-regulated by drought and abscisic acid (ABA) treatments [26], [27]. In contrast, expression of *OsDhn1* is most strongly induced by drought, although induction by cold, high salinity and ABA has also been demonstrated [25].

In a previous study, over-expression of *Arabidopsis DREB1B/CBF1* was found to up-regulate the expression of *OsDhn1* in transgenic rice plants [25], suggesting activation of the *OsDhn1* promoter through a drought-responsive element(s). Similarly, up-regulation of the *Dhn8* gene was observed in transgenic bahia grass plants (*Paspalum notatum* Flugge cv. Argentine) transformed with a CaMV35S-*HsDREB1A* fusion construct containing a DREB gene from *Hordeum spontaneum* [28], and up-regulation of *HvDhn8* and *TaCor410* in transgenic barley and wheat plants was also seen following constitutive or drought-inducible over-expression of either *TaDREB2* or *TaDREB3* [29]. However, *cis*-acting promoter elements responsible for the constitutive expression and stress-inducible activation of either *TaCor410* or *TaCor410*-like genes have not been identified. Moreover, while several TFs are reported to regulate *Cor410* gene expression, it is not known which specific TFs are likely to be most important for stress-inducible activation of *Cor410*.

In this work, the promoter of the *TdCor410b* stress-inducible gene was isolated from durum wheat and used for identification of functional DRE/CRT *cis*-elements *via* a transient expression assay. TFs that bind the critical functional CRT element were isolated and their ability to activate the *TdCor410b* promoter was evaluated. Molecular modeling was used to investigate the nature of protein-DNA binding interactions between different types of ERF/DREB TFs and promoter elements.

## Materials and Methods

Nucleotide sequences reported in this work have been deposited in GenBank under Accession numbers JN681186 (*TdCor410b*), JN681187 (*TdERF6*), JN681188 (*TaERF6*), JN681189 (*TaERF4a*), JN681190 (*TaERF4b*), JN681191 (*TaERF5a*), JN681192 (*TaERF5b*) and JN681193 (*HvERF4*).

### Promoter cloning and plasmid construction

The full-length coding region of the *TaCor410* cDNA (GenBank accession L29152) was isolated by PCR using a cDNA library obtained from spikes of drought-stressed wheat (*Triticum aestivum* L cv. Chinese spring) as a template. The *TaCor410* cDNA was used as a probe to screen a BAC library prepared from genomic DNA of *Triticum durum* cv. Langdon [30], as previously described [31]. The selected BAC clone (#661 E9) was used as a template for isolation by PCR of the *T. durum* homolog of *TaCor410* (*TdCor410*), with primers derived from the coding region of the *TaCor410* cDNA. The *TdCor410b* promoter sequence was identified through sequencing of the BAC clone. A 2685 bp long promoter region containing a full-length 5′-untranslated region of *TdCor410b* was cloned into the pMDC164 vector [32] as described [31] and the resulting construct was designated pTdCor410b-GUS. *TdCor410b* promoter deletions were generated by PCR using AccuPrime™ Pfx DNA polymerase (Invitrogen, Mulgrave, Victoria, Australia) and the *TdCor410b* promoter as a template. PLACE software (http://www.dna.affrc.go.jp/PLACE/signalup.html) was used to predict DRE/CRT elements in the *TdCor410b* promoter region, and forward primers were designed so as not to interrupt potential *cis*-elements. Promoter deletions were cloned into the pMDC164 vector and used in transient expression assays described below.

An artificial promoter was generated by substitution of the functional CRT element in the shortest active deletion of the *TdCor410b* promoter (263 bp), with three repeats of the GCC-box (AGCCGCC). A tandem of GCC-boxes was added to the sequence of the forward PCR primer and the artificial promoter was generated by PCR. Together with the full length *TdCor410b* promoter, the artificial promoter was used in transient expression assays to test activation properties of ERFs and molecular variants of TaERF4a *in planta*.

The coding regions for *TaDREB2*, *TaDREB3*, *TaERF4*, *TaERF4a*, *TaERF5a*, *TdERF6*, *GFP* and *GUS* were cloned into the pENTR-D-TOPO vector (Invitrogen). The cloned inserts were verified by sequencing, subcloned into the pUbi vector [29] and used for transformation of wheat cell cultures. pUbi-GFP and pUbi-GUS plasmids were used as negative and positive controls, respectively, and for quantification of the efficiency of biolistic bombardment in the transient expression assays described below.

### Transient expression assay

A transient promoter activation assay, based on co-bombardment of promoter-GUS fusion constructs with pUbi-TF constructs, was performed using a suspension cell culture of *T. monoccocum* L. initiated from roots [33]. Cell suspensions were grown in 100 ml of liquid medium (½-strength Murashige-Skoog (MS) medium supplemented with 2 mg/L of 2,4-dichlorophenoxyacetic acid (2,4-D) in the dark at 25°C, and were sub-cultured weekly. Cell suspensions were harvested on the sixth day following subculture by sieving in a laminar-flow hood and approximately 1 ml of the cell material was spread over a piece of Whatman filter paper to form a circle of 3.5 cm in diameter. This material was incubated on ½-strength MS + 2,4-D+300 mM sucrose for 2 h prior to bombardment. The concentration of each plasmid sample was adjusted to 0.5 µg/µl, then 5 mL each of a plasmid containing TF coding sequence and a plasmid containing promoter regions were mixed and co-precipitated with 1 µl of 3 M sodium acetate (pH 4.8) and 15 µl 100% (v/v) isopropanol. The DNA precipitates were recovered by centrifugation (13,000× g, 4°C, 15 min). The pellet was washed twice in 75% (v/v) ethanol and dried in a laminar-flow hood. The pellet containing a mixture of plasmid DNAs was dissolved in 10 µl MilliQ water and used for coating 0.6 µm gold particles [34]. Microprojectile bombardment was performed using the Biolistic PDS-1000/He Particle Delivery System (Bio-Rad, Hercules, CA, USA). Bombardment conditions were 1100 psi, with a 15 mm distance from the macrocarrier launch point to a stopping screen and a 60 mm distance from the stopping screen to the target plant material. The distance between the rupture disk and the launch point of the macrocarrier was 12 mm. The pre-cultured cell suspensions were bombarded on growth media containing 150 mM sucrose, and transformed cells were incubated on the same growth media in the dark at room temperature for 40–48 h. GUS staining solution was prepared as described [35], except that 20% (v/v) methanol was added to the solution before use. Filters containing the transformed cells were transferred to Petri dishes and 1.3 ml of GUS staining solution was pipetted under the filter paper so as not to disturb the circle of cell suspension. The stained cells were incubated overnight at 37°C. GUS activity was determined by counting the number of blue cells (foci) using a Leica DC 300F stereomicroscope (Leica Microsystems GmbH, Nussloch, Germany). For each combination of constructs, 3 – 4 independent bombardments were performed. The pUbi-GFP construct was used to determine the efficiency of bombardment. Statistical analyses were performed by one-way ANOVA (GenStat 9.0).

### Plant transformation and analysis of transgenic plants

Two vectors were generated, where the 2×35S promoter was excised using the *Hind*III and *Kpn*II restriction sites from the pMDC32 vector [32], and replaced with either 2,685 or 275 bp long fragments of the *TdCor410b* promoter. These vectors were designated as pCor410H and pCor410H2, respectively. The coding region of *TaDREB3* cDNA [36] was cloned into pCor410H and pCor410H2 and the resultant constructs were transformed into barley (*Hordeum vulgare* L. cv. Golden Promise), using *Agrobacterium*-mediated transformation [29]. Transgene integration was confirmed by PCR using a forward primer from the 3′ end of the promoter and a reverse primer from the 5′ end of the *nos* terminator. The basal level of activity of the *TdCor410b* promoter fragments in leaves of transgenic T_0_ lines was determined by northern blot hybridization analysis using coding region of TaDREB3 cDNA as a probe. To analyse activity of the long and short versions of the promoter in transgenic barley plants, seedlings of each of three selected transgenic lines and three control wild type plants were grown together in 10-inch pots containing 2.5 kg of standard coco peat potting mix in a growth chamber (24°C/50% relative humidity (day) and 18°C/80% (night), with a 12 h photoperiod). For the drought induction assay, plants were well watered for three weeks and then water was withheld. Leaf samples were collected on the last day of watering; two further samples were collected at 7 and 10 days after the cessation of watering. Relative soil water content measured using a Fieldscout spectrometer (Spectrum technologies Ltd., Illinois, USA) indicated soil water contents of 48 (for well-watered), 7, and 3% (v/v), respectively. For the induction of the *TdCor410b* promoter by cold, plants were grown for three weeks before being transferred to a cold cabinet (BINDER GmbH, Tuttlingen, Germany) maintained at 4°C. Leaf samples were collected before the cold treatment and after 2, 8 and 24 hours of incubation at 4°C. For the analysis of promoter inducibility by wounding, the leaves of 3-week-old seedlings were mechanically wounded with a fine metal brush and harvested at 0, 0.5, 1, 4, 8, and 24 hours after wounding. Leaves from three biological replicates were used for RNA isolation and Q-PCR analysis.

### Preparation of cDNA libraries and isolation of TFs using a Y1H screen

*TaDREB2* (Acc. DQ353852) and *TaDREB3* (Acc. DQ353853) were previously isolated from a bread wheat (*T. aestivum* cv. Chinese Spring) endosperm cDNA library (WENDL) [36].

A barley cDNA library (BCG) was prepared from floral tissues/flag leaf of cold-tolerant barley (cv. Haruna Nijo) under cold/frost stress. Plants were grown to anthesis in a growth chamber set to the following conditions: four weeks at 20°C (day)/8°C (night) with a 10 h photoperiod; four weeks at 21°C (day)/10°C (night) with a 12 h photoperiod; then 22°C (day)/12°C (night) with a 14 h photoperiod. At anthesis, plants were moved to a frost chamber. Flag leaves and whole spikes were sampled when the temperature at floret height (i) fell to 4°C; (ii) had been held at the minimum temperature of −5.5 °C for 2 h; and (iii) had returned to 4°C. The RNA was pooled from each time point (30% from the first time point, 50% from the second time point and 20% from the third time point), so that the contribution from each time point comprised equal amounts of RNA from 12 individual heads from each of three plants.

A wheat cDNA library (WHSL) was prepared from flag leaves and spikes of an Australian drought-tolerant bread wheat (*T. aestivum* cv. RAC875), that had been subjected to high temperatures under both well-watered and drought stress conditions. Plants were grown in well-watered conditions to anthesis in a growth chamber 22°C (day)/10°C (night) with a 14 h photoperiod). At flowering, plants were subjected to seven days of heat stress, where on each day, the day-time temperature was gradually increased to 40°C (10 min at each of 24, 27, 29, 30, 32, 34, 36°C), held at 40°C for a further 4 h, then lowered to 28°C for 2 h and returned to 22°C for the remainder of the day and overnight. Watering was withheld from the second day, and plants showed signs of water deficiency from the fourth day. On the first day of the heat stress treatment (well-watered), and again on the fourth and seventh days (drought-stressed), samples of flag leaf and spike (at different stages of development) were collected at two time points; as soon as the temperature reached 40°C, and again after a further 3.5 h at 40°C. Tissue samples were collected from five plants in total. A mixture of equal amounts of total RNA from each plant was used for cDNA library preparation.

The WENDL, WHSL, and BCG cDNA libraries were screened with baits constructed from five repeats of a GCCGAC (CRT1) core element, or three repeats of a 16 bp long *TdCor410b* promoter fragment, TTCCG**GCCGAC**ACGCT (CRT2, bold type indicating the GCCGAC core element) [36]. Twenty four positive clones were analysed for each library/bait pair.

### Transcriptional activation and DNA binding assays in yeast

The coding regions of selected representatives from each of the three cloned subfamilies of *ERF*s, *TaERF4a*, *TaERF5a* and *TdERF6*, were amplified by PCR using primers with additional *Eco*RI and either *Bam*H1 (*TaERF4a* and *TdERF6*) or *Pst*I (*TaERF5a*) restriction sites. The amplified fragments were cloned into the respective restriction sites of the pGBKT7 vector and the resultant constructs were transformed into yeast (*Saccharomyces cerevisiae* strain AH109). Yeast transformants carrying the plasmids were selected on synthetic defined (SD) (-Trp) medium and replica-plated to SD2 (-Trp, -His) medium. The ability of transformants to grow on SD2 medium suggested the presence of a native activation domain in the *ERF*.

### Construction of three-dimensional (3D) models of AP2 DNA-binding domains of TaERF4a, TaERF5a and TaDREB3

Three-dimensional models were constructed by comparative (homology) modeling that relies on applying spatial restraints derived from a structural template [37]. Templates for the AP2 domains of TaERF4a, TaERF5a and TaDREB3 were identified *via* 3D-PSSM [38], LOMETS [39], MUSTER [40] and the Structure Prediction Meta-server [41]. The most suitable template for all three AP2 domains was identified to be the AP2 of AtERF1 [Protein Data Bank (PDB) accession number 1 gcc, chain A, here designated as 1 gcc:A] from *Arabidopsis thaliana* [42]. The 1 gcc:A structure was solved by NMR in complex with the 5′-GCTA**GCCGCC**AGC-3′ *cis*-element [42]. Full-length sequences of TaERF4a, TaERF5a and TaDREB3 were analysed by ProDom [43] to determine domain arrangements and the boundaries of the AP2 domains. After the domain boundaries of the TaERF4a, TaERF5a and TaDREB3 AP2 domains were identified, the sequences were aligned with 1 gcc:A by PROMALS3D [44]. The aligned sequence pairs were further investigated by Hydrophobic Cluster Analysis (HCA) [45] to confirm that the secondary structures of the proteins remained undisturbed. As the 1 gcc:A 3D structure was elucidated in the presence of a double stranded *cis*-element, these data gave us the opportunity to model the wheat AP2 domains in complex with their respective *cis*-elements identified in the current work. Hence, AP2 of TaERF4a was modelled with GCCGAC, AP2 TaERF5a with GCCGCC and GCCGAC, and TaDREB3 with ACCGAC and GCCGAC. The individual *cis*-elements were generated *via* the Sybyl 8.0 suite of programs (Tripos International, St. Louis, MO, USA) and were minimized with a Tripos force field. The aligned template and target sequences with their respective *cis*-elements were further used as input parameters to generate 3D models of the TaERF4a, TaERF5a and TaDREB3 AP2 domains (62, 62 and 63 residues, respectively), using Modeller 9v7 [37], and running the Fedora 12 operating system on a Linux station. The most optimal models with the lowest value of the Modeller 9v7 objective function and the most favourable discrete optimized protein energy scoring parameters were chosen from 50 models for optimisation with a Tripos force field (Sybyl 8.0). A Ramachandran plot of the optimized AP2 models indicated that 100% of residues were in the most favoured, additionally allowed and generously allowed regions, when excluding the Gly and Pro residues, indicating that protein stereochemistry was satisfactory. The overall G-factors (estimates of stereochemical parameters) evaluated by PROCHECK [46], were −0.23, −0.13, −0.12 and −0.19 for 1 gcc:A, TaERF4a, TaERF5a and TaDREB3, respectively. The Z-score values deduced from Prosa2003 [47], reflecting combined statistical potential energy, were -5.5, −6.1, and−5.9 and −6.3 for 1 gcc:A, TaERF4a, TaERF5a and TaDREB3, respectively. The rmsd values, between 1 gcc:A and TaERF4a, TaERF5a and TaDREB3 (superpositions of total of 62 residues in each case), determined with the PyMol (http://www.pymol.org) ‘super’ algorithm were 0. 24 Å, 0.25 Å and 0.25 Å in the Cα positions, respectively. The electrostatic potentials were calculated with the Adaptive Poisson-Boltzmann Solver (the dielectric constants of solvent and solute were 80 and 2, respectively) (http://apbs.sourceforge.net/) implemented in PyMol as a plugin, and mapped onto the protein molecular surfaces that were generated with a probe radius of 1.4 Å. Molecular graphics was generated with PyMol (http://www.pymol.org).

### Phylogenetic analysis of TFs containing the AP2 domain

The amino acid sequences of 32 AP2 domain-containing plant TFs including those of 13 ERFs with C-terminal repressor motifs, were aligned with AtERF1 (1 gcc:A from *A. thaliana*) [42] and a phylogenetic tree, based on a crude distance measure, was generated using PROMALS3D [48]. The tree was visualised using TreeView [49]. The TF sequences included in this analysis were TaDREB2 (Acc. ABC86563), TaDREB3 (Acc. ABC86564), TaDREB6 (Acc. AAX13289), GhDREB (Acc. AAQ08000), TmCBF12 (Acc. ABW87011), BjDREB1B (Acc. ABX00639), AtDREB1A (Acc. BAA33434), AtDREB2A (Acc. BAA33435), GmERF3 (Acc. ACD47129), GmERF4 (Acc. ACE76905), AtERF1 (Acc. AB008103), NtWRAF1 (Acc. BAF48803), NtWRAF2 (Acc. BAF48804), HvERF1 (Acc. ADO21119), OsBIERF1 (Acc. AAV98700), CaERFLP1 (Acc. AAS20427), TaERF3 (Acc. ABQ52687), AtERF3 (Acc. NP_175479), AtERF4 (Acc. NP_188139), AtERF7 (Acc. NP_188666), AtERF8 (Acc. NP_175725), AtERF9 (Acc. NP_199234), AtERF10 (Acc. NP_171876), AtERF11 (Acc. NP_174159), AtERF12 (Acc. NP_174158) and NsERF3 (Acc. BAA97123).

### Quantitative PCR

Q-PCR analysis of the expression of the *TdCor410b* and *ERF* genes in different tissues and under several stresses were performed as described [50]. Absolute expression of genes of interest (Table S1) were normalised against three control genes and were converted to measurements of (normalised) copy number per µg of total RNA used in the reverse transcription reaction. The cDNA tissue series were prepared from different tissues of *T. aestivum* (cv. Chinese spring). The stress cDNA series for Q-PCR analysis was prepared from three to four leaves that were collected from each of 2 – 4 independent 6-week-old plants of either *T. aestivum* (cv. RAC875) and/or *T. durum* (cv. Langdon), subjected to each of the following stresses: drought (samples were collected from well-watered plants, wilted plants under strong drought (soil volumetric water content of 3%), and two weeks after re-watering); cold stress at 4°C (samples were collected following 0, 1, 4, 24, and 48 hours of cold stress); and wounding with a fine metal brush (samples of *T. aestivum* were collected at 0, 1, 3, 7 and 24 h after wounding, samples of *T. durum* were collected at 0, 0.25, 0.5, 1, 1.5, 2, 3, 4, and 7 h after wounding).

## Results

### Identification of functional DRE/CRT cis-elements in the TdCor410b promoter, and confirmation of their involvement in response to different stresses

A homolog of the *TaCor410* gene, and regulatory sequences starting 2,685 bp upstream of the translational start codon, were isolated from a BAC library prepared from *Triticum durum* cv. Langdon [30]. The cloned gene contained a single intron of 111 bp. An alignment of the deduced protein to TaCor410 homoeologs and similar proteins from rice and barley demonstrated that the gene product from *T. durum* has the greatest amino acid sequence similarity to TaCor410b (only a single residue difference; **Figure S1**), and was therefore designated as TdCor410b.

**Figure 1:**
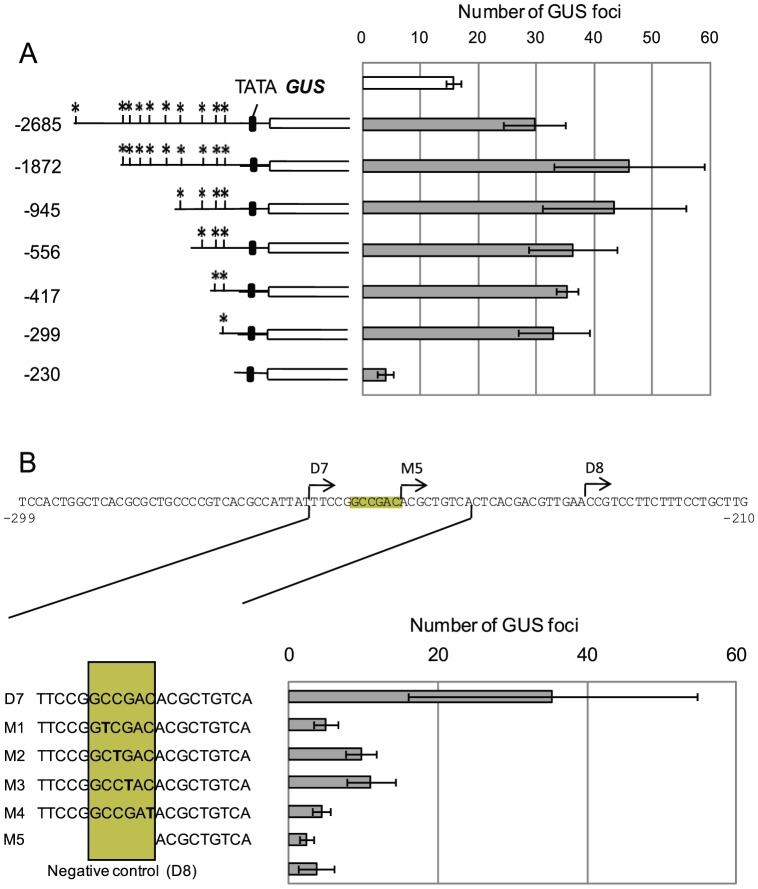
Identification of functional DRE/CRT element in the *TdCor410b* promoter using transient expression assay. (A), Identification of functional drought-responsive DRE/CRT elements by 5′ deletion analysis of the *TdCor410b* promoter, using *trans*-activation of the *GUS* reporter gene in a transient expression assay. The full-length *TdCor410b* promoter and six promoter deletions were linked to the *GUS* reporter gene and co-transformed *via* particle bombardment into cell suspension cultures with either pUbi-GFP (negative control) or pUbi-TaDREB3 (transcription activator). A schematic representation of the 5′ terminal deletions of the promoter fused to the *GUS* gene is shown in the left part of the figure: asterisk (*) denotes the predicted DRE/CRT site. A negative control (basal levels of full-length promoter activity) is shown at the top of the right panel as an empty box. Error bars represent standard deviation (P<0.05) for 3 – 4 independent measurements. (B) Influence of point mutations in the identified functional CRT element on *TdCor410b* promoter activation, as demonstrated by a transient expression assay. D7 denotes a −263 promoter deletion containing the non-mutated CRT element (positive control), D8 and M5 denote promoter deletions without the CRT element (negative controls), and M1–M4 denote the D7 deletion with different single base pair substitutions to T.

Ten DREs/CRTs/LTREs, two ABREs, and several putative abiotic stress-related MYC and MYB responsive elements [51] were identified in the 2,685 bp promoter region of *TdCor410b* using PLACE software [52], [53]. Of the ten predicted DRE/CRT/LTRE elements, five were of the GCCGAC type and three were of the ACCGAC type (**Figure S2**). No GCC-box was identified in the promoter region of *TdCor410b*. It has previously been demonstrated that the promoters of *TaCor410*-like genes from rice, barley and wheat can be activated in transgenic plants through over-expression of DREB proteins [25], [28], [29]. We therefore used TaDREB3 to activate 5′ truncated segments of the *TdCor410b* promoter in transient expression assays, with the aim of identifying functional *cis*-element(s). Mixtures of equal amounts of pUbi-GFP (negative control) or pUbi-TaDREB3 with the pTdCor410b-GUS plasmid(s), containing deletions in the *TdCor410b* promoter, were used to co-transform a cell suspension culture of *T. monoccocum*. Deletions of the promoter were generated based on putative *cis*-acting elements at −1872, −945, −556, −417, −299, and −230 bp (**Figure S2**). Each of these deletions, except deletion −945, decreased the number of putative DRE/CRT elements by one, thus creating the opportunity to evaluate individual elements for functionality (**Figure 1A**). A basal level of activity of the *TdCor410b* promoter was detected when the negative control was used for co-transformation instead of TaDREB3. Cell cultures transformed with −1872, −945, −556, −417, and −299 deletions in the promoter region showed similar induction of GUS expression over basal levels, of between 2.1 and 2.9-fold. However, the −230 bp promoter deletion could not activate the reporter gene, indicating that the *TdCor410b* promoter is regulated by TaDREB3 through the putative DRE/CRT element located between −299 and −230 bp (**Figure 1A**). The element responsible for basal levels of promoter activity was evidently located on the same segment of the promoter, because the -230 bp long deletion could provide only about a quarter of the basal activity of the full-length promoter. The sequence of the DRE/CRT element in this region recognised by TaDREB3 is TTCCG**GCCGAC**ACGCT (the bold type indicates the GCCGAC core element). The GCCGAC core element is referred to as a cold-responsive element that functions in *Arabidopsis* as the G**GCCGAC**AT element [5], [54] and in barley as the (G/a)(C/t)CGAC element [6]. The GCCGAC core element differs from the originally identified DRE element, T**ACCGAC** [55], [56] **,** used for the isolation of TaDREB3 [36], in the first base pair of the core element. It was shown previously that both GCCGAC and ACCGAC are responsible for activation of promoters *via* cold and drought [5], [54]–[56]. However, we have found that the GCCGAC and ACCGAC elements have different protein-binding specificities, and for this reason we designate these elements as CRT and DRE types, respectively.

Several single bp mutations introduced into the core sequence of the mapped functional CRT element in a −263 bp deletion of the *TdCor410b* promoter were used in transient expression assays to verify functionality of the identified *cis*-element (**Figure 1B**). Activation of GUS fused to each of the mutant fragments was compared with activity of the D7 (−263 bp) (positive control) and D8 (−230 bp) (negative control) deletions after co-bombardment with the pUbi-TaDREB3 construct. Each of the four tested mutations strongly decreased the activity of the −263 promoter deletion. However, substitution of the second C and last C of the core element for T was the most critical for DNA-protein binding. These mutations decreased the activity of the −263 deletion to the level of the negative control (**Figure 1B**). The *HvDhn8* promoter sequence available from the NCBI databases (Acc. AF043093) was compared with that of *TdCor410b*. The position and adjacent sequences of the mapped CRT element were conserved (**Figure S3A**). Co-bombardment of pHvDhn8-GUS and pUbi-TaDREB3 constructs resulted in a 6-fold activation of the promoter with TaDREB3 compared with the negative control (**Figure S3B**).

Unfortunately, activation of promoter fragments by stresses such as drought and wounding cannot be tested using a transient expression assay. Analysis of transgenic barley plants expressing *TaDREB3* driven by 2,685 bp and 275 bp fragments of the *TdCor410b* promoter revealed the presence of basal levels of promoter activity, and inducibility of both promoter fragments by cold, drought and wounding (**Figure S4**). This analysis confirmed that activation of the *TdCor410b* promoter by stress, and even in the absence of stress, occurred providing the CRT element immediately proximal to the TATA box was retained.

### Isolation of TFs using a CRT element as bait

The core sequence GCCGAC repeated five times (CRT1), or three repeats of a fragment of the *TdCor410b* promoter containing the GCCGAC core sequence, (TTCCG**GCCGAC**ACGCT) (CRT2), were used in a yeast one-hybrid system to screen three separate prey libraries. These were WENDL, a library prepared from wheat un-stressed endosperm, WHSL, a library prepared from drought/heat-stressed wheat flag leaf and spikes, and BCG, a library prepared from cold/frost-stressed barley floral tissues and flag leaf. The WENDL cDNA library was previously used for the isolation of DREB proteins and TFs that are not induced by stress [36]. Because the *TaCor410b* gene is expressed in early grain/endosperm in the absence of stress, we searched for potential up-stream activators of this gene in the WENDL library. The barley cDNA library (BCG) was used because a cDNA library from wheat tissue subjected to cold/frost treatment was not available. The amounts of RNA from various time intervals in this library reflect our attempt to enrich the library with early-responsive genes and genes responsive to temperatures below zero.

The coding sequences of six ERFs and one DREB were isolated in Y1H screens from WENDL: *TaERF5a*, *TaERF4a*, *TaERF5b*, *TaERF6* and *TaDREB2*; from WHSL: *TaERF4a* and *TaERF4b*; from BCG: *HvERF4*. All listed TFs were isolated using the CRT1 element. *TaERF4a* and *HvERF4b* were also isolated in screens with the CRT2 element, as well as clones containing partial cDNA sequences of *TaERF5b* and *TaERF6*. An Expressed Sequence Tag (EST) encoding the 5′ end of the *TaERF5b* cDNA was identified from the NCBI databases (Acc. CA728064), and the full-length sequence of *TaERF5b* cDNA was isolated from WHSL cDNA using nested PCR. No complementary ESTs have been deposited in the NCBI databases for the *TaERF6* cDNA. However, the intron-less gene of the *TaERF6* orthologue from *T. durum* was identified in BAC clone #191 I19, using a segment of the coding region of *TaERF6* as a probe. The full-length coding region of this gene, designated *TdERF6*, was used to make a construct for transient expression assays.

In total, seven different AP2-domain-containing TFs were isolated, only one of these (*TaDREB2*) belonging to the DREB family. The remaining six TFs encoded *TaERF4a, TaERF4b, HvERF4, TaERF5a, TaERF5b*, and *TaERF6*, all belonging to subfamilies of the ethylene-responsive element (GCC-box) binding TFs (*EREBP* ***s*** or *ERF*s). *TaERF5a, TaERF5b,* and *TaERF6* had been isolated previously using the GCC-box as bait from the same cDNA libraries (unpublished data). However, no *TaERF4*-like TFs have been isolated with the GCC-box from any cDNA library.

Absence of the *TaDREB3* cDNA among isolated clones can be explained by low abundance of this cDNA [29], and an insufficient number of analysed clones to identify sequences with low abundance.

### Phylogenetic analysis of TFs isolated in Y1H screens and DNA binding and activation properties of ERFs

**Figure 2:**
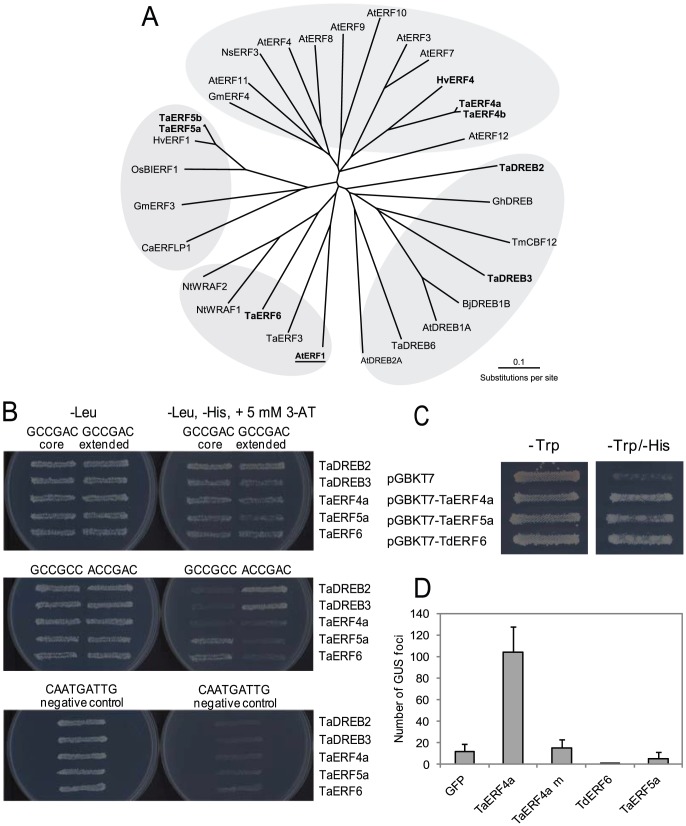
Wheat TFs isolated in Y1H screens and their properties. (A) An unrooted radial phylogenetic tree of AP2-domain containing TFs from monocotyledonous and dicotyledonous plant species. Amino acid sequences of 32 proteins were aligned with ProMals3D (44) and branch lengths were drawn to scale. Grey shading indicates distinct branches of ERF and DREB TFs. Two-letter prefixes in the sequence identifiers indicate species of origin (Ta  =  *Triticum aestivum*; Hv  =  *Hordeum vulgare*; Os  =  *Oriza sativa*; Gm  =  *Glycine max*; At  =  *Arabidopsis thaliana*; Bj  =  *Brassica juncea*; Gh  =  *Gossypium hirsutum*; Nt  =  *Nicotiana tabacum*; Ns  =  *Nicotiana sylvestris*; Ca  =  *Capsicum annuum*). Protein accession numbers are specified in the Materials and Methods. TFs isolated in this work are shown in bold. The *Arabidopsis* AtERF1 TF was used for construction of 3D models of the AP2 domains of TaERF4a, TaERF5a and TaDREB3, and is shown in bold and underlined. (B) Specificity of recognition of known stress-responsive *cis*-elements by ERF and DREB TFs detected *via* a Y1H assay. Growth of yeast on selective medium (-Leu, -His,+5 mM 3-AT) indicates protein-DNA interaction. The *cis*-element CAATGATTG of the HD-Zip class II TF was used as a negative control. (C) Demonstration of activator properties using ERFs in a Y1H assay. The presence of their own activation domains in the representatives from each subfamily of ERFs supports the activation of the yeast genes and consequent growth of yeast on the selective (-Leu, -Trp, -His, -Ade) medium. (D) Regulation of *TdCor410b* promoter activity by representatives of each isolated subfamily of ERFs. TFs were tested in a transient expression assay in a wheat cell culture. The pTdCor410b-GUS construct was co-bombarded with pUbi-GFP (GFP; negative control), pUbi-TaERF4a (TaERF4a), pUbi-TaERF4a mutated in the ERF-associated amphiphilic repression (EAR) motif (TaERF4a m), pUbi-TaERF6 (TaERF6), and pUbi-TaERF5a (TaERF5a), and GUS expression in the cultures was quantified (n = 4±SD (P<0.05)).

Phylogenetic analysis (**Figure 2A**) indicated an evolutionary relationship between wheat TFs isolated in the Y1H screen and known homologues from other plant species [21], [57]–[62]. The unrooted tree of 32 TFs containing AP2 domains from mono- and dicotyledonous species was constructed to establish a phylogenetic relationship among the individual proteins (**Figure 2A**). It was also important to establish phylogenetic relationships with AtERF1 from *Arabidopsis* (in bold characters and underlined), as this protein was used as a template for molecular modeling of the AP2 domains of TaERF4a, TaERF5a and TaDREB3. The full-length sequence of the selected mono- and dicotyledonous ERF and DREB proteins clustered into four independent branches, highlighting their functional roles (**Figure 2A**). This clustering is in agreement with their DNA binding selectivity as demonstrated by Y1H assays (**Figure 2B**). The analysis of selectivity of binding of *cis*-elements confirmed that all tested TFs from wheat could bind the CRT (GCCGAC) core element. We could not detect differences for any of the tested factors between their binding to the CRT1 (GCCGAC) and CRT2 (TTCCG**GCCGAC**ACGCT; the bold type indicates the GCCGAC core element) sequences. Thus, the core element itself may be sufficient to confer specificity of binding, and the influence of adjacent sequences may be minimal. Y1H assays also established that the DREB TFs could bind the DRE (ACCGAC) motif, but could not bind the GCC-box (GCCGCC). As expected, TaERF5a and TaERF6 could interact with the GCC-box, but could not bind the DRE motif. Surprisingly, TaERF4a could bind neither the GCC-box nor DRE, binding only to CRT (**Figure 2B**).

Representatives from each subfamily of isolated ERFs, *TaERF4a*, *TaERF5a* and *TaERF6*, were tested in yeast for the presence of activation domains and their ability to activate a yeast reporter gene. All three proteins behaved as activators (**Figure 2C**). Each of the proteins, when fused to the binding domain of the yeast GAL4 TF, could activate a downstream reporter gene and consequently support yeast growth on selective medium (**Figure 2C**).

The full-length coding regions of *TaERF4a*, *TaERF5a* and *TdERF6* were cloned into the pUbi vector and examined for their ability to activate the *TdCor410b* promoter in a wheat suspension cell transient expression assay. Here, we found that only *TaERF4a* activated the full-length promoter of the *TdCor410b* gene, and this activation was about 6 – 7 fold higher than the basal level of promoter activity (**Figure 2D**). *TaERF5a* and *TaERF6* could not activate the *TdCor410b* promoter, but either partially or totally inhibited the basal activity of the promoter (**Figure 2D**). These inhibitory effects of *TaERF5a* and *TaERF6* were observed in several independent experiments.

Mutations that were introduced into a predicted ERF-associated amphiphilic repression (EAR) motif of TaERF4a strongly decreased promoter activation. The mutations consisted of substitutions of four conserved residues in the EAR motif to Ala (D164A, L165A, N166A, and P169A; **Figure S6B**). *TdCor410b* promoter activity was reduced to basal levels by these mutations (**Figure 2D**).

### Expression patterns of TaCor410b and ERFs in different tissues and under different stress conditions

**Figure 3:**
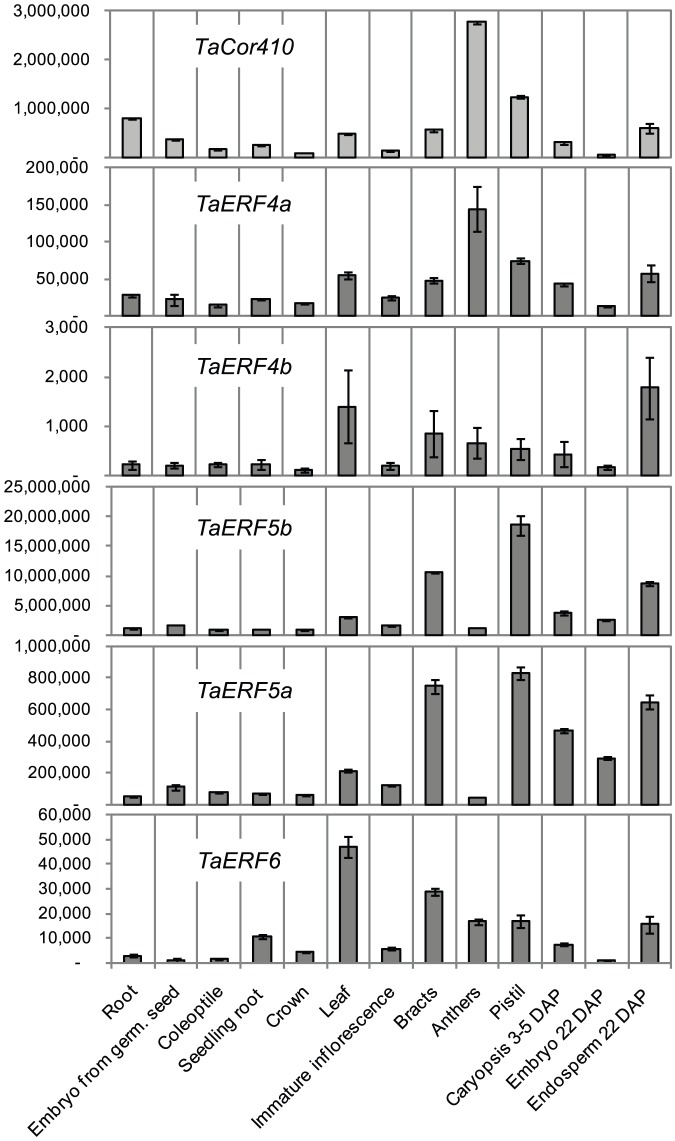
Expression of *TaCor410b* and five *ERF* genes in a variety of wheat tissues in the absence of stress. Levels of expression were detected by Q-PCR and are shown as normalised transcription levels in arbitrary units (n = 4± SD (P<0.05)).

Spatial expression patterns of *TaCor410b* and five *ERF* genes isolated in the Y1H screen were analysed using Q-PCR. In the absence of stress, expression of *TaCor410b* was detected in all tissues analysed, with strongest expression in anthers and pistils shortly before fertilization. *TaDREB3*, which weakly activated *TaCor410b* in transgenic wheat [29] and the *TdCor410b* promoter in transient assays, was also expressed in reproductive tissues [29]. Co-expression analysis of the ERFs and *TaCor410b* in the absence of stress showed that the pattern of expression of *TaERF4a* closely correlated with the expression of *TaCor410b* in all tested tissues, thus making the *TaERF4a* gene the best candidate for regulation of *TaCor410b* in the absence of stress (**Figure 3**). The expression pattern of *TaERF4b* showed very little correlation with the expression patterns of *TaERF4a* or *TaCor410b*, but closely resembled that of *TaERF6* (**Figure 3**). The close homologues, possibly homoeologues, *TaERF5a* and *TaERF5b*, had very similar expression patterns, although expression of *TaERF5b* was consistently about 20-fold higher than that of *TaERF5a*.

**Figure 4:**
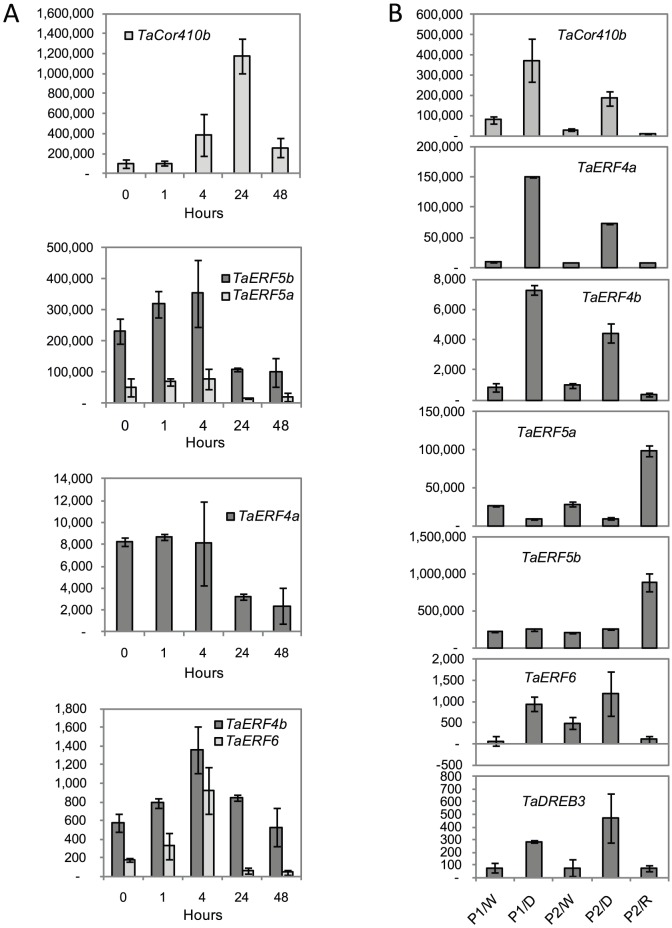
Stress-inducible expression of *TaCor410b* and *ERF/DREB* genes in leaves of 4-week old wheat seedlings. (A) Expression of *TaCor410b* and five *ERF* genes during cold (4°C) stress. (B) Expression of *TaCor410b*, *TaDREB3* and five *ERF* genes in leaves of two different plants (P1 and P2) under well-watered conditions (W), drought (D), and two-weeks after re-watering (R). Levels of expression were detected by Q-PCR and are shown as normalised transcription levels in arbitrary units (n = 4 ± SD (P<0.05)).

Cold stress, imposed as a constant treatment at 4°C, strongly induced *TaCor410b* by about eleven-fold (**Figure 4A**). Expression of the gene started to increase within several hours, and reached maximum levels after 24 h of plant exposure to cold, but returned to near-basal levels after 48 h (**Figure 4A**). The wheat *ERF* genes and barley *HvERF4* (**Figure 4A and S** **5**), as well as *TaDREB3* and *TaDREB2* [29] showed a weak to mild induction by cold during the first four hours of stress exposure, with expression levels declining with prolonged treatment. The induction of *ERFs* and *DREBs* by cold stress always preceded induction of the downstream *TaCor410b* gene (**Figure 4A**).

Under stringent drought conditions, where leaf wilting was observable and volumetric water content in soil was ≤3%, *TaCor410b* was up-regulated approximately four-fold (**Figure 4B**). *TaCor410b* expression returned to normal levels after re-watering and two weeks of recovery. Under these drought stress conditions, similar induction of expression, followed by a return to normal levels after re-watering and recovery, was also observed for *TaERF4a*, *TaERF4b*, *TaERF6*, and *TaDREB3.* By contrast, the expression of *TaERF5a* decreased under stringent drought conditions, while expression of *TaERF5b* was not responsive to water deficit. Increased expression of both genes, by 2.5 – 3 fold, was observed following re-watering and recovery from drought (**Figure 4B**).

**Figure 5:**
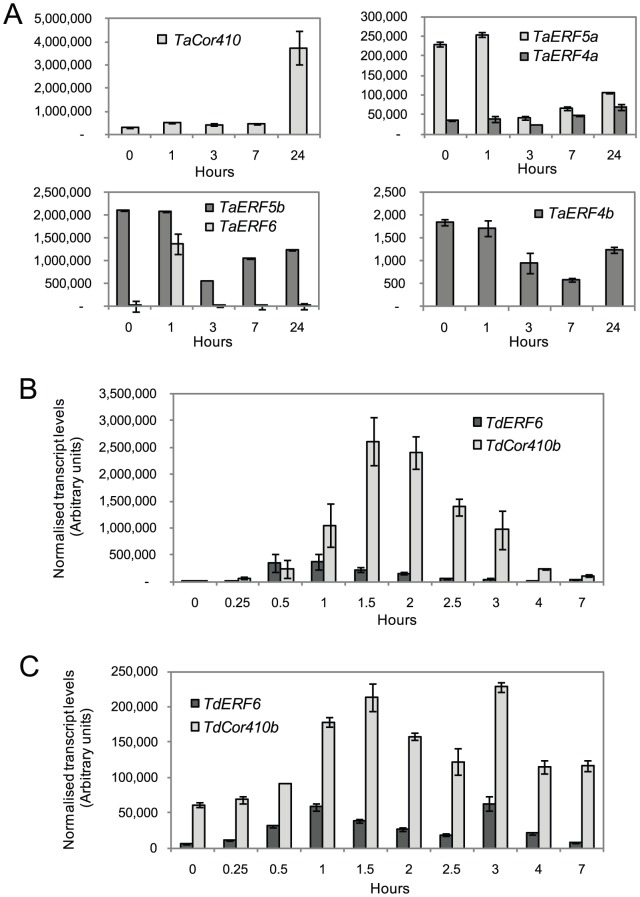
Expression of *Cor410b* and *ERF* genes in leaves and grain of bread and durum wheat subjected to mechanical wounding. (A) Expression of *TaCor410b* and *TaERF* genes in leaves of bread wheat plants following wounding. Levels of expression, detected by Q-PCR, are shown as normalised transcription levels in arbitrary units. (B) Expression of *TdCor410b* and *TdERF6* following wounding in leaves of durum wheat plants at flowering. (C) Expression of *TdCor410b* and *TdERF6* wheat grains following wounding, with the wounding being applied at 8–15 days after pollination. Values are means (± SD (P<0.05)) of 3 measurements.

Wounding of leaves of a three-week old wheat seedling resulted in 1.5-fold activation of *TaCor410b* RNA levels one hour after the wounding. After 24 hours, the levels of expression were 12-fold higher than those in a control leaf (**Figure 5A**). The expression patterns of all tested *ERFs* except for *TaERF6* were very similar, showing a strong reduction in expression at three hours after wounding, and partial or complete restoration to normal expression levels after 24 h. *TdERF6* induction in response to wounding in leaves and developing grain preceded that of *TdCor410b* (**Figure 5B and 5C**), and the same temporal relationship between *TaERF6* and *TaCor410b* was also observed in leaves of bread wheat (**Figure 5A**).

### Domain organisation and structural alignments of AtERF1 (1 gcc:A) with AP2 domains of TaERF4a, TaERF5a and TaDREB3

**Figure 6:**
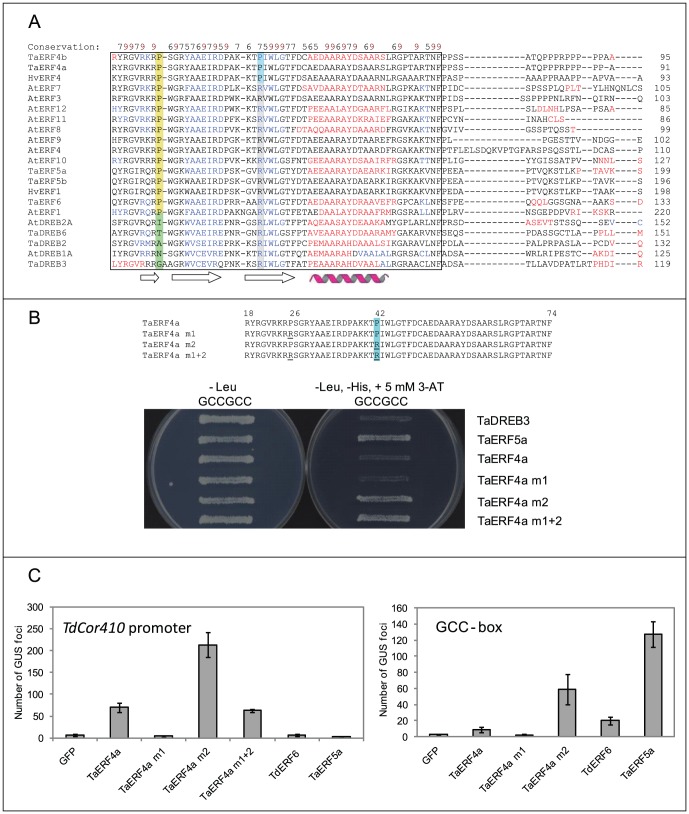
Key residues of AP2 domains that underlie selectivity of *cis*-elements binding, and regulation of the *TdCor410b* promoter activity. (A) Multiple sequence alignment of selected AP2 domains using PROMALS3D (44). Representative sequences are coloured according to predicted secondary structures (red: α-helix, blue: β-strand). The black box indicates the boundaries of the AP2 domains. The positions of highly conserved Pro residues in the ERF sequences and of variable non-proline residues in the DREB sequences are highlighted in yellow and green, respectively. The positions of two Pro residues conserved in selected cereal ERF sequences are highlighted in cyan, while the positions of the corresponding Arg residues are highlighted in grey. Consensus of secondary structure elements indicates the position of β-sheets (black arrows) and of an α-helix (purple). The degree of conservation of residues is shown above the sequences by black and brown numbers with a conservation index of 5 and higher. (B) Influence of conserved proline residue substitutions in the AP2 domain of TaERF4a on recognition of the GCC-box. TaDREB3 was used as a negative control and TaERF5a as a positive control of interaction with the GCC-box. Mutation of Pro26 to Arg26 (underlined) has no influence on interaction of the TaERF4a variant with the *cis*-element. Mutation of Pro42 to Arg42 (underlined and boxed in blue) lead to restoration of interaction and consequent growth of yeast on the selective (-Leu, -His, + 5 mM 3-AT) medium. (C) Regulation of the activity of the *TdCor410b* promoter and of the artificial promoter with substitution of the CRT element for a tandem of three GCC-boxes by representatives of each isolated ERF subfamily, and variants of TaERF4a with mutations in the AP2 domain. TFs were tested in a transient expression assay in a wheat cell culture. Either pTdCor410b-GUS or 3×GCCbox-GUS constructs were co-bombarded with pUbi-GFP (GFP; negative control), pUbi-TaERF4a (TaERF4a), pUbi-TaERF4a mutated at Pro26 (TaERF4a m1), pUbi-TaERF4a mutated at Pro42 (TaERF4a m2), pUbi-TaERF4a mutated at Pro26 and Pro42 (TaERF4a m1+2), pUbi-TaERF6 (TaERF6), or pUbi-TaERF5a (TaERF5a).

The AP2 domain (or GCC-box binding domain) of AtERF1 from *Arabidopsis* (PDB accession 1 gcc:A) was used for comparative structural modelling and analysis of ERF and DREB TFs isolated in our studies, due to the presence of this domain (of approximately 62 residues) in both subfamilies of TFs. Structural alignment of 32 AP2 domain-containing sequences provided information about the conservation of the AP2 domains at the amino acid level within selected TFs. Analysis indicated that the sequences could be divided into two major groups, based on conservation of a Pro residue following Arg152 in 1 gcc:A; Arg152 makes close interactions with the coding strand of a DNA element [42]. While this Pro residue was conserved in all ERF sequences included in the alignment (**Figure 6A**), a highly variable residue was present in the corresponding position of the analysed DREB sequences (regions highlighted in green and yellow, respectively, in **Figure 6A**). Further examination of the alignment revealed that the ERF sequences could be sub-divided into two additional subgroups. The first subgroup comprised members of the subfamily of TaERF4a-like proteins, which contained Pro42 in the TPI motif in position 42, whereas all other examined ERFs contained Arg in the corresponding position (regions highlighted in cyan and grey in **Figure 6A**). This analysis suggested the significance of Arg, Pro and other adjacent residues that may play roles in a recognition selectivity of the GCC-box by ERFs (**Figure 6A** **)**. Of critical importance was the observation that Pro42 found in the TaERF4a-like proteins occurred exclusively in monocotyledonous species, as confirmed by the analysis of 501 sequences (data not shown) through the ConSurf server [63].

### Molecular modeling of the AP2 domains of TaERF4a, TaERF5a and TaDREB3 to reveal selectivity of binding of cis-elements

The suitability of AtERF1 from *A. thaliana* (designated here as 1 gcc:A) as a template for modelling was confirmed through searches using PsiPred [64], SAM-T08 [65], STRIDE [66], DSSP [67], PROMALS3D [48] and Robetta [68]. The sequence of 1 gcc:A [69] was aligned with TaERF4a, TaERF5a and TaDREB3, whereby care was taken that the positions of secondary structures of proteins remained undisturbed. The positional sequence identity and similarity between AtERF1 (1 gcc:A) and TaERF4a, TaERF5a and TaDREB3, determined by an Epprofile algorithm [70], were 40% and 55%, 31% and 50%, and 38% and 53%, respectively. The sequence identity between 1 gcc:A and TaERF5a was close to the so-called ‘twilight zone’ case and this fact emphasized a complexity of modeling [71]. Pairwise alignments between the template and the target sequences, TaERF4a, TaERF5a and TaDREB3, indicated that there was one single-residue deletion (corresponding to Asn167 in1 gcc:A) in all three alignments (data not shown).

**Figure 7:**
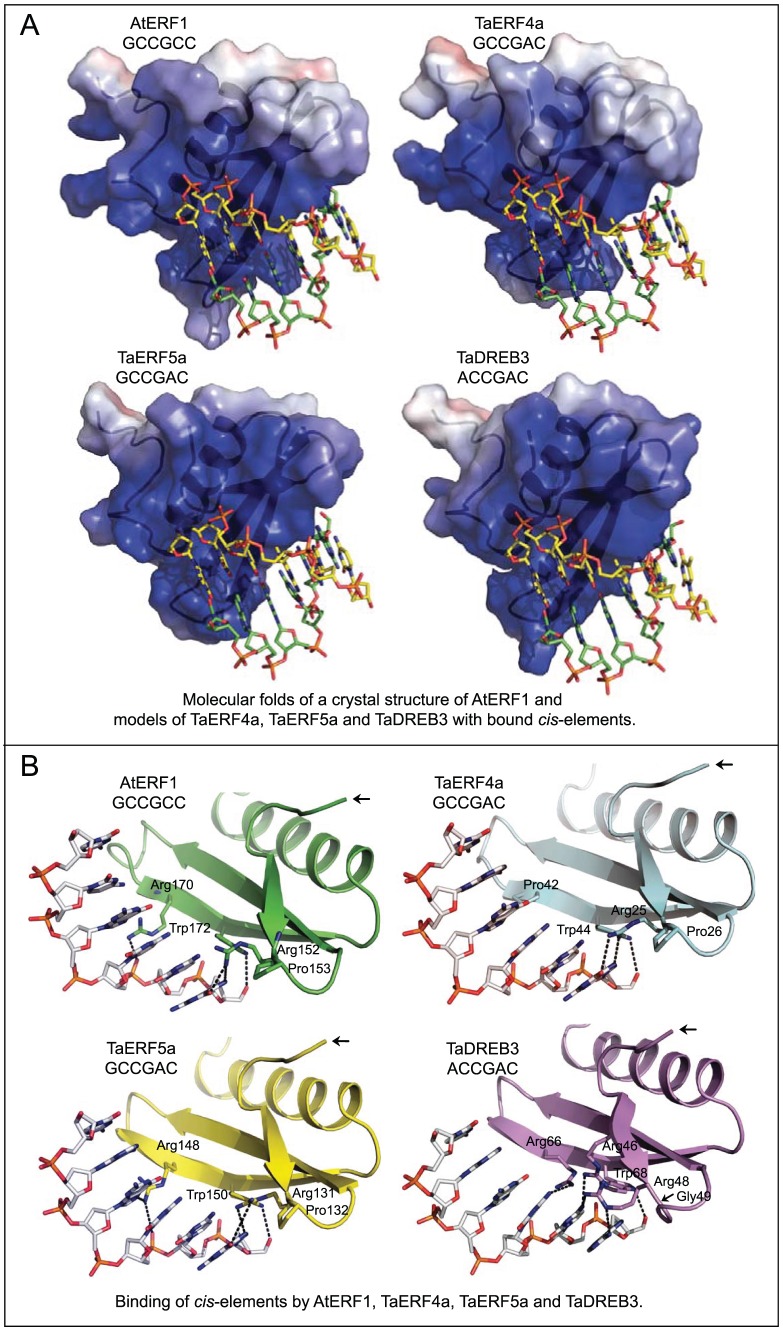
Molecular models of AP2 domains in complex with *cis*-elements. (A) Molecular surface morphologies of the AP2 domains of AtERF1, TaERF4a, TaERF5a and TaDREB3 TFs in complex with *cis*-elements. White, blue and red patches on protein surfaces indicate electro-neutral, electropositive and electronegative patches; the charged patched are contoured at ±5 kT/e. Double-stranded DNA sequences of the *cis*-elements (GCCGCC/GGCGGC, GCCGAC/GTCGGC and ACCGAC/GTCGGT) are indicated by sticks, where the coding and complementary strands are shown in green and yellow atomic colours, respectively. (B) Molecular folds of the AP2 domains of AtERF1, TaERF4a, TaERF5a and TaDREB3 TFs in complex with *cis*-elements. Ribbon representations show the disposition of secondary structure elements, where anti-parallel strands carry amino acid residues that mediate contacts between individual *cis*-elements and the AP2 domains. The ribbons are coloured in green (AtERF1), cyan (TaERF4a), yellow (TaERF5a) and magenta (TaDREB3). The black arrows point to the NH_2_-termini of the AP2 domains. The coding strands of *cis*-elements GCCGCC, GCGGAC and ACCGAC are shown as stick models and are coloured in atomic colours. The interacting residues in the AP2 domains are also shown as sticks, and are coloured in green (AtERF1), cyan (TaERF4a), yellow (TaERF5a) and magenta (TaDREB3). Distances of ≥3.4 Å between the contacting residues (Arg and Trp) and *cis*-elements are indicated by dotted lines. The positions of respective Pro or Gly residues, adjacent to the contacting Arg residues, are also indicated. The interplay of these residues within the structures suggested that structural rigidity or flexibility could impact upon selectivity of binding of individual *cis*-elements.

Analyses through PROCHECK [46] and Prosa2003 [47] indicated that the 3D models were reliable and that the stereochemistry of protein structures was satisfactory. The sequence identities between the TaERF4a, TaERF5a and TaDREB3 AP2 domains were within similar ranges, and therefore it was not surprising to detect similar protein folds, as well as a high degree of conservation of residues in all 3D models (**Figure 7A**). It is evident in **Figure 7B** that all three TFs contained an α-helix and a three-stranded anti-parallel β-sheet. This type of architecture is characteristic of a global ‘alpha and beta protein’ class, which bind DNA, according to SCOP protein classification [72]. Calculations of electrostatic potentials revealed the presence of a highly positively-charged depression within the structure of the AP2 domains, where the double stranded *cis*-elements would be expected to bind (**Figure 7A**). As the molecular models of the AP2 domains of TaERF4a, TaERF5a and TaDREB3 were generated in the presence of their respective *cis*-elements, we could envisage how the individual DNA hexamers bound within the AP2 grooves, and precisely how structural determinants underlied the recognition selectivity of the respective *cis*-elements (**Figure 7A and 7B**). Molecular modeling revealed that the coding strands, rather than the complimentary strands, of DNA elements were bound through a series of highly conserved residues exposed on the two longer anti-parallel β-sheets, and that conserved Arg and Trp residues mediated the contacts between *cis*-elements and the AP2 domains in all instances (**Figure 7B**). It was of note that, from all the residues within the AP2 domains, the conservation of two Pro residues in TaERF4a, TaERF5a and HvERF4 was most obvious, as well as the presence of variable residues in DREBs at the end of a short β-sheet and in the middle of the β-sheet (**Figures 6A** **and** **7B**). These comparisons suggested that the β-sheets in the ERF or DREB AP2 domains can flex to a higher or lesser degree, due to the presence or absence of Pro, and that this β-sheet flexibility could affect the overall architecture of the AP2 domains, or more or less favourably re-orient individual *cis*-elements. This could lead to tighter or weaker binding of *cis*-elements by individual AP2 domains. Comparisons of TaDREB3 in complex with GCCGAC and ACCGAC indicated that Arg48, which is positioned next to Gly49 (**Figures 6A** **and** **7B**), had significant flexibility and could reach out and mediate close contacts with both *cis*-elements. By contrast, flexibility of Arg131 in TaERF5a (a factor that binds both GCCGCC and GCCGAC) could be severely restricted due to the presence of neighbouring Pro132. The question then arises as to why the GCCGCC *cis*-element is only recognised by the AP2 domain of TaERF5a and not by TaDREB3? Our modeling studies indicated that the recognition selectivity of TaDREB3 could be decided by several structural features. Firstly, the overall length of the protein segment spanning Gly49 to Arg66 (16 residues, compared to 15 residues in the ERF AP2 domains) might be of importance and, secondly, the specific environment around Arg48 and Arg66 might be critical, preventing binding of the GCC-box by TaDREB3. On the other hand, the environment around Arg131 in the AP2 of TaERF5a (iso-positional to Arg48 in AP2 of TaDREB3), and a shorter β-sheet region comprising 15 residues between Pro132 and Arg148 (iso-positional to the Gly49-Arg66 region in TaDREB3's AP2), would allow binding of both *cis*-elements GCCGCC and GCCGAC. However, the length of the β-sheet segment that forms a DNA binding region in TaERFs cannot be the only structural requirement that determines binding of the GCC-box, because TaERF4a does not bind to GCC-box elements. In the AP2 domain of TaERF4a, the presence of the two relatively closely positioned Pro residues could restrict flexibility of the β-sheet, thus preventing interactions with the GCC-box. Conversely, binding of GCCGAC by the AP2 domain of TaERF4a could be favourable, because an amino group in the purine ring of adenine could mediate productive interactions with AP2 (**Figure 7B**).

### Site-directed mutagenesis of amino acid residues to determine recognition selectivity of the AP2 domain of TaERF4a

The molecular model of the AP2 domain of TaERF4a, and its comparison with the AP2 models of TaERF5a and TaDREB3 in complex with a variety of *cis*-elements (**Figure7**), allowed generation of variant proteins of the AP2 domain of TaERF4a with potentially modified selectivity for binding the GCC box (GCCGCC) (**Figure6**). Through site-directed mutagenesis, we mutated each of the two conserved Pro residues to create a Pro26Arg mutant (TaERF4a m1), a Pro42Arg mutant (TaERF4a m2), and a Pro26Arg+Pro42Arg double mutant (TaERF4a m1+2; **Figure 6B**). The double mutant was designed to modify flexibilities of cognate β-sheets through side-chain residue variations, to mimic properties of the respective β-sheets and disposition of residues within TaDREB3.

Complete restoration of binding to the GCC-box by the AP2 domain of TaERF4a was obtained by replacing Pro42 with Arg42 (TaERF4a m2). The yeast GCC-box bait strain grew on the selective medium when TaERF4a m2 was expressed, while this was not the case for TaERF4a m1 (**Figure 6B**). The ability of the double mutant, TaERF4a m1+2, to grow on the selective medium was likely due only to the Pro42Arg mutation (**Figure 6B**). The expression of wild type TaERF4a could not support growth of the yeast GCC-box bait strain under the same selective conditions (**Figure 6B**). These data were further confirmed using transient expression assays in wheat cell cultures. An artificial promoter, containing three repeats of the GCC-box was weakly activated by wild type TaERF4a. This promoter was not activated by TaERF4a m1, but was strongly activated by TaERF4a m2 (**Figure 6C**). The functionality of the artificial promoter was confirmed by activation of this promoter with TaERF5a and TaERF6 TFs. These findings demonstrated the activation behaviour of the latter two ERFs *in planta* and confirmed our observations in yeast (**Figure 2C**). Surprisingly, the wild type *TdCor410b* promoter was also strongly activated by TaERF4a m2, but was not activated by TaERF4a m1 and was only weakly activated by TaERF4a m1+2. In contrast to TaERF4a m2, neither TaERF5a nor TaERF6 TFs were able to activate the wild type *TdCor410b* promoter in the transient expression assay.

## Discussion

Several important *cis*-elements involved in regulating promoters of stress-inducible genes in plants have been identified and studied previously. These studies, however, have focussed on the model plant *Arabidopsis* [4], [5], instead of more agrononically-relevant, monocot species. Furthermore, little has been done to understand the complexity of regulation of particular promoter elements by TFs *in planta*. For example, can a single *cis*-element be recognised by multiple TFs? Is the same *cis*-element regulated differently under different environmental conditions? Is basal constitutive expression of a stress-responsive gene regulated through the same or different *cis*-element(s) in the promoter? Can strength and/or specificity of protein-DNA interactions be modulated by genetically engineered variants of existing TFs? These and related questions have been addressed in the current work using the *TdCor410b* promoter.

### The TdCor410b promoter

In this study we have focused on DRE/CRT elements in the *TdCor410b* promoter. DRE/CRT elements are involved in abiotic-stress responses, including drought and cold, and are known to be bound mostly by one class of TFs, namely, DREB/CBFs. We predicted ten potential DRE/CRT/LTR elements in the *TdCor410b* promoter. However, activation by the *TaDREB3* TF was confirmed only with the CRT element closest to the potential TATA-box. Our results show that regulation of the stress-inducible *TdCor410b* promoter is complex and involves the participation of several different types of AP2 domain-containing TFs. These different TFs use a single, ‘promiscuous’ CRT element with a core sequence GCCGAC. The CRT element may be involved in cold-induced activation of *TdCor410b* by TaDREB3 or other DREB/CBF proteins. It is possible that other upstream CRT(s) could become functional, at least partially, if the primary element was lost or mutated. Alternatively, other DREB/CBFs may target other DRE/CRT elements within the same promoter.

Basal activity of the *TdCor410b* promoter was mapped to a -299 bp fragment of the promoter, suggesting that the same single CRT *cis*-element may be responsible for both constitutive activity and inducible activation of the *TdCor410b* promoter. This hypothesis was confirmed when several single-base mutations were introduced into the mapped element (**Figure 1B**). Furthermore, a comparison of sequences of the *TdCor410b* and *HvDhn8* promoters revealed a high level of conservation of the position of the GCCGAC elements and of the adjacent sequences in both promoters. Activation of the *HvDhn8* promoter by TaDREB3 was demonstrated in transgenic barley plants with constitutive overexpression of *TaDREB3* [29], as well as in this study using transient assays (**Figure S3**). Furthermore, barley plants were stably transformed with *TaDREB3* under the regulation of the 2,567 bp and 275 bp regions of the *TdCor410b* promoter. Analysis of transgenic lines demonstrated that both promoter regions drove basal levels of *TaDREB3* expression, and both were activated by cold, drought and wounding (**Figure S4**). These results defined the role of the CRT element proximal to the TATA box as a universal element, which could regulate *TdCor410b* promoter activity under optimal growth conditions and in response to a variety of abiotic stresses.

### TdCor410b activation

To better understand the mechanism of promoter activation, we isolated TFs that bound to the *TdCor410b* promoter, using the GCCGAC element (CRT1) as bait in Y1H screens of cDNA libraries prepared from both un-stressed and stressed wheat or barley tissues. Seven different AP2 domain-containing TFs were isolated in the screen. Surprisingly, only one, *TaDREB2*, belonged to the DREB subfamily. The other six TFs belonged to the ERF subfamily of the AP2 domain family. Genes from the DREB/CBF subfamily have been reported to play a critical role in responses of plants to abiotic stress through DRE/CRT elements within the core motif (A/G)CCGAC [54], [56], [73]. In contrast, the ERF subfamily members, formally known as EREBPs, are mainly involved in responses to pathogens and wounding through recognition of the GCC-box A**GCCGCC** (bold type indicating the core GCC element) [7]–[13], [58], [74]–[82]. The ability of a number of ERFs to also interact with the GCCGAC sequence has been demonstrated [12], [77], [83], [84] using Electrophoretic Mobility Shift Assays, an artificial system where aberrant binding may occur. In our study, a Y1H assay and plant cell culture analyses were used to determine functional binding of TFs to *cis*-elements. The Y1H assay revealed *in vivo* interactions for all three types of identified wheat ERFs with the GCCGAC element. However, only two types of ERFs were able to bind the GCC-box and, as expected, neither interacted with the ACCGAC element (**Figure 2B**). The functionality of such interactions was confirmed by the ability of TaERF4a to activate the *TdCor410b* promoter in transient expression assays (**Figure 2D**). In contrast to TaERF4a, the other two types of ERFs did not activate the *TdCor410b* promoter. Substitution of the CRT element for a three-fold repeat of the GCC-box in the same promoter, however, led to activation (**Figure 6C** **)**.

### Mode of action of TaERF4

The most abundant independent clones isolated in the Y1H screen were homologues of *TaERF4a*, *TaERF4b* and *HvERF4*. All three genes belong to the same subfamily of ERF factors that have homologies to *AtERF3* and *AtERF4* from *Arabidopsis* [58], [62], and to *ERF3* from *Nicotiana sylvestris* [59], [60] (**Figures 2A and S** **6**). *AtERF3, AtERF4* and the tobacco *ERF3* are all believed to function as repressors, and their gene products contain a C-terminal ERF-associated amphiphilic repression (EAR) motif (L/F)DLN(L/F)(X)P, that has more recently been found in other families of TFs [85]. TaERF4a, TaERF4b and HvERF4 also contain the EAR motif, but our functional analyses indicated that they function as activators of promoter activity rather than repressors. The substitution of four key amino acid residues in the EAR motif for alanine residues strongly decreased the promoter activation properties of TaERF4a in both Y1H and transient expression assays (**Figure 2D**). In contrast to TaERF4a, TaERF4b and HvERF4, subfamily members from tobacco and *Arabidopsis* contain Arg42 instead of Pro42 in the AP2 domain, and were shown to strongly interact with the GCC-box [58], [60]. Alignment and conservation analysis through the ConSurf server revealed that Pro42 can only be found in ERF sequences of monocotyledonous plants. Although we have demonstrated that Pro42 changed the specificity of protein-DNA binding of ERF4 subfamily members, the biological significance of Pro at this position in monocotyledonous plants remains to be determined. Other TFs containing the EAR domain have also been shown to act as transcriptional activators [86]. Although the mechanism of such activation has not been explained, it has been suggested that indirect regulation through repression of repressors may occur.

It is likely that *TaERF4a* functions as a specific regulator of the *TdCor410b* promoter, because transcript expression of *TaERF4a* and *TaCor410b* was highly correlated (**Figure 3**).

### Structure of TaERFs

Three-dimensional models of the AP2 domains of TaERF5a, TaERF4a and TaDREB3 were constructed based on the DNA-binding domain of AtERF1 in complex with the 5′-GCTA**GCCGCC**AGC element. The mutual interplay of residues within the secondary structure elements of the AP2 domains that form a β-sheet, could impact upon structural rigidity or flexibility of AP2 domains, and may affect DNA binding selectivity. The overall shape variability and disparity in surface electrostatic potentials among individual AP2 domains of ERF and DREB TFs, could also contribute to differences in binding selectivity of *cis*-elements.

Our attempt to restore the binding ability of TaERF4a to the GCC-box through site-directed mutagenesis (**Figure 6B**) needs to be discussed in connection with recent molecular dynamics simulations of TFs [87]. Wang *et al*. [87] reported that the significance of the Arg150, Arg152, Arg170 and Trp172 residues in the AP2 domain of AtERF1 for binding the GCC-box differs between AtERF1, AtERF4 and AtCRT/DREB1 [85]. Arg150, Arg152, Arg170 and Trp172 are *iso*-positional to Arg23, Arg25, Pro42 and Trp44 in AP2 of TaERF4a; our modelling indicated that only the two conserved Arg23 and Arg25 residues directly contacted the first G in **G**CCGAC in the coding strand of the DNA element, as well as two G bases in the complementary strand, GTC**GG**C. Therefore, these two residues mediate primary DNA binding for **G**CCGAC/GTC**GG**C. The Pro42 residue in the AP2 domain of TaERF4a does not interact with the GCCGAC element. Modeling also indicated that mutation of Pro42 to Arg would create a variant form of TaERF4 that could potentially bind base C of GCCG**C**C, and we were able to demonstrate this experimentally in our study (**Figure 6B**).

Thus, structural comparisons of the AP2 domains of TaERFs and TaDREBs, in complex with *cis*-elements, identified the specific variations in amino acid residues that affected flexibility of the secondary structure. These variations lead to differences in recognition selectivity of *cis*-elements by TaERF and TaDREB DNA binding domains.

### Interactions between ERFs

**Figure 8:**
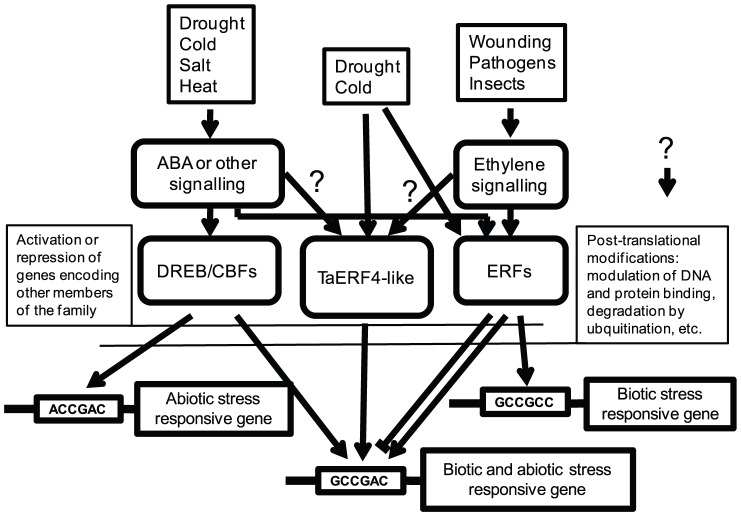
A schematic representation of the regulation of abiotic and biotic stress-responsive genes by ERFs and DREBs/CBFs, through three types of stress-responsive *cis*-acting elements.

Although both TaERF6 and TaERF5a behaved as activators in yeast, they appeared to compete for CRT binding with endogenous TaERF4-like or DREB/CBF proteins in wheat cell cultures, and were unable to activate the *TdCor410b* promoter. However, TaERF6 and TaERF5a were both able to activate a modified promoter, where the CRT element was substituted for the GCC-box. Synchronised expression of the *TaERF6* and *TdCor410b* genes in response to wounding suggests that TaERF6 may be a candidate for wounding-induced *TdCor410b* promoter activation, and this could occur *via* the CRT element. Other wounding-inducible or tissue-specific TFs or modifying enzymes may be required to assist TaERF6 activation of the *TdCor410b* promoter. In our transient expression assay, TaERF6 down-regulates the basal activity level of the *TdCor410b* promoter, which indicates there is a TaERF6 protein-promoter interaction, albeit a negative one, *in planta*. Further investigation will be required to understand if additional TFs function as part of an activation complex involving TaERF6, or as passive repressors of genes interacting with other CRT elements, during pathogen attack and/or during plant recovery after abiotic stress (**Figure 8**). Additionally, TaERF6 may directly or indirectly act as a passive repressor of two other subfamilies of ERF genes. Partial repression of transcription was observed for members of ERF4 and ERF5 subfamilies shortly after activation of TaERF6 by wounding (**Figure 5A**). The closest published homologues of TaERF6 are the wound-inducible ERFs, WRAP1 and WRAP2, from tobacco [61], which were not reported to be induced by abiotic stresses. Here we found that TaERF6 was weakly induced by both cold and drought, evidence that TaERF6-like TFs are involved in abiotic stress regulation in monocotyledonous species.

*TaERF5a* and *TaERF5b* were found to be close homologues/orthologues of rice *OsBIERF1*, which shows moderate expression in the absence of stress and is induced by a number of biotic and abiotic stresses including cold, salt and drought [75]. No clear influence of TaERF5a on *TdCor410b* promoter activity was detected in wheat cell culture transient expression assays. *TaERF5a* was down-regulated in leaves of drought-stressed plants, whereas no changes in expression were detected for *TaERF5b*. Therefore, these proteins are unlikely to be active positive regulators of *TaCor410b* in response to drought. However, TaERF5b strongly activated the artificial *Cor410b* promoter *via* the GCC-box in our transient assays, suggesting this ERF may be an ethylene-regulated activator.

### Conclusion

We suggest that *TaCor410* genes are likely to be regulated by ERF and DREB/CBF TFs through a single CRT (GCCGAC) element. Stress-responsive induction of *TdCor410b* indicated that a complex interplay of ERF and DREB/CBF TFs takes place, which may also involve other TFs and modifying factors. The best candidate for driving constitutive activity and drought-inducible activation of *TdCor410b* promoter was TaERF4a. The exact role, if any, of two other types of ERFs in *TdCor410b* promoter regulation requires further investigation. TaERF4a possessed properties that were atypical of other ERFs investigated in this study, including unusual DNA-binding specificity and specific transcriptional activation.

## Supporting Information

Figure S1 Multiple sequence alignment of protein sequences of TdCor410b and products of homoeologous genes from bread wheat, and reported homologues from barley and rice: Wcor410 (Acc. AAA20189), Wcor410b (Acc. AAB18201), Wcor410c (Acc. AAB18202), HvDHN8 (Acc. AAD022259), OsDHN1 (Acc. AAV49032). Identical amino acid residues are in yellow boxes, conserved residues are in blue boxes, and similar residues are in green boxes.
(EPS)

Figure S2 The sequence of the *TdCor410b* promoter with predicted CRT/DRE/LTRE elements. The putative TATA-box is in bold and underlined, the predicted elements are in grey boxes, the functional element is in a grey box and underlined. First bp of each promoter deletion used in promoter mapping is marked with a black box. Names and sizes (bp) of promoter deletions are shown above the black boxes.
(EPS)

Figure S3 Comparison of the *TdCor410b* and *HvDHN8* promoters. (A) Pair-wise alignment of nucleotide sequences of the *TdCor410b* and *HvDHN8* promoters. Computer-predicted cis-elements common for both promoters are in transparent boxes; sequence of the functional cis-element is marked with *. The putative TATA-box and translational start are in bold. (B) Basal activity of the *TdCor410b* and *HvDhn8* promoters (1) and activity induced by overexpression of *TaDREB3* (2). The promoter-GUS construct was co-bombarded in the wheat suspension cell culture with either the pUbi-GFP (1) or pUbi-TaDREB3 (2) constructs.
(EPS)

Figure S4 Activation of a -275 bp and -2,685 bp long promoter fragments by wounding, cold and drought in transgenic barley plants detected by Q-PCR.
(EPS)

Figure S5 The Q-PCR analysis of HvERF4 expression in leaves and roots of barley plants subjected to cold (4oC).
(EPS)

Figure S6 Sequence alignment of AP2 domains and EAR motifs of TaERF4a-like proteins. (A) A multiple sequence alignment of thirteen AP2 domains of the ERF sequences using PROMALS3D (41). The positions of highly conserved Pro residues in the ERF sequences are highlighted in yellow and the positions of three Pro residues conserved in the selected cereal ERF sequences are highlighted in cyan. (B) The conserved regions of the COOH-terminal EAR sequence underlying the importance of four conserved residues Asp, Leu, Asn and Pro, are in pink.
(EPS)

Table S1 List of primers used for Q-PCR.
(DOCX)

## References

[1] Gao MJ, Allard G, Byass L, Flanagan AM, Singh J (2002) Regulation and characterization of four CBF transcription factors from Brassica napus. Plant Mol Biol 49: 459–471. 12090622 10.1023/a:1015570308704

[2] Kizis D, Pages M (2002) Maize DRE-binding proteins DBF1 and DBF2 are involved in *rab17* regulation through the drought-responsive element in an ABA-dependent pathway. Plant J 30: 679–689. 12061899 10.1046/j.1365-313x.2002.01325.x

[3] Maruyama K, Sakuma Y, Kasuga M, Ito Y, Seki M, et al (2004) Identification of cold-inducible downstream genes of the *Arabidopsis* DREB1A/CBF3 transcriptional factor using two microarray systems. Plant J 38: 982–993. 15165189 10.1111/j.1365-313X.2004.02100.x

[4] Sakuma Y, Maruyama K, Osakabe Y, Qin F, Seki M, et al (2006) Functional analysis of an *Arabidopsis* transcription factor, DREB2A, involved in drought-responsive gene expression. Plant Cell 18: 1292–1309. 16617101 10.1105/tpc.105.035881 PMC1456870

[5] Stockinger EJ, Gilmour SJ, Thomashow MF (1997) *Arabidopsis thaliana* CBF1 encodes an AP2 domain-containing transcriptional activator that binds to the C-repeat/DRE, a cis-acting DNA regulatory element that stimulates transcription in response to low temperature and water deficit. Proc Natl Acad Sci USA 94: 1035–1040. 9023378 10.1073/pnas.94.3.1035 PMC19635

[6] Xue GP (2002) An AP2 domain transcription factor HvCBF1 activates expression of cold-responsive genes in barley through interaction with a (G/a)(C/t)CGAC motif. Biochim Biophys Acta 1577: 63–72. 12151096 10.1016/s0167-4781(02)00410-4

[7] Brown RL, Kazan K, McGrath KC, Maclean DJ, Manners JM (2003) A role for the GCC-box in jasmonate-mediated activation of the *PDF1.2* gene of Arabidopsis. Plant Physiol 132: 1020–1032. 12805630 10.1104/pp.102.017814 PMC167040

[8] Guo HW, Ecker JR (2004) The ethylene signaling pathway: new insights. Curr Opin Plant Biol 7: 40–49. 14732440 10.1016/j.pbi.2003.11.011

[9] Hao DY, Ohme-Takagi M, Sarai A (1998) Unique mode of GCC box recognition by the DNA-binding domain of ethylene-responsive element-binding factor (ERF domain) in plant. J Biol Chem 273: 26857–26861. 9756931 10.1074/jbc.273.41.26857

[10] Nishiuchi T, Suzuki K, Kitajima S, Sato F, Shinshi H (2002) Wounding activates immediate early transcription of genes for ERFs in tobacco plants. Plant Mol Biol 49: 473–482. 12090623 10.1023/a:1015553232309

[11] Ohme-Takagi M, Shinshi H (1995) Ethylene-inducible DNA binding proteins that interact with an ethylene-responsive element Plant Cell. 7: 173–182. 10.1105/tpc.7.2.173 PMC160773 7756828

[12] Park JM, Park CJ, Lee SB, Ham BK, Shin R, et al (2001) Overexpression of the tobacco *Tsi1* gene encoding an EREBP/AP2-Type transcription factor enhances resistance against pathogen attack and osmotic stress in tobacco. Plant Cell 13: 1035–1046. 11340180 10.1105/tpc.13.5.1035 PMC135557

[13] Xiong LM, Schumaker KS, Zhu JK (2002) Cell signaling during cold, drought, and salt stress. Plant Cell 14: S165–S183. 12045276 10.1105/tpc.000596 PMC151254

[14] Canella D, Gilmour SJ, Kuhn LA, Thomashow MF (2010) DNA binding by the Arabidopsis CBF1 transcription factor requires the PKKP/RAGRxKFxETRHP signature sequence. Biochim Biophys Acta 1799: 454–462. 19948259 10.1016/j.bbagrm.2009.11.017

[15] Xu ZS, Xia LQ, Chen M, Cheng XG, Zhang RY, et al (2007) Isolation and molecular characterization of the *Triticum aestivum* L. ethylene-responsive factor 1 (TaERF1) that increases multiple stress tolerance. Plant Mol Biol 65: 719–732. 17874224 10.1007/s11103-007-9237-9

[16] Yi SY, Kim JH, Joung YH, Lee S, Kim WT, et al (2004) The pepper transcription factor *CaPF1* confers pathogen and freezing Tolerance in Arabidopsis. Plant Physiol 136: 2862–2874. 15347795 10.1104/pp.104.042903 PMC523348

[17] Close TJ, Kortt AA, Chandler PM (1989) A cDNA-based comparison of dehydration-induced proteins (dehydrins) in barley and corn. Plant Mol Biol 13: 95–108. 2562763 10.1007/BF00027338

[18] Mundy J, Yamaguchi-Shinozaki K, Chua NH (1990) Nuclear proteins bind conserved elements in the abscisic acid-responsive promoter of a rice *rab* gene. Proc Natl Acad Sci USA 87: 1406–1410. 2137613 10.1073/pnas.87.4.1406 PMC53484

[19] Danyluk J, Houde M, Rassart E, Sarhan F (1994) Differential expression of a gene encoding an acidic dehydrin in chilling sensitive and freezing tolerant Gramineae species. FEBS Lett 344: 20–24. 7910142 10.1016/0014-5793(94)00353-x

[20] Ganeshan S, Vitamvas P, Fowler DB, Chibbar RN (2008) Quantitative expression analysis of selected *COR* genes reveals their differential expression in leaf and crown tissues of wheat (*Triticum aestivum* L.) during an extended low temperature acclimation regimen. J Exp Bot 59: 2393–2402. 18508811 10.1093/jxb/ern112 PMC2423658

[21] Danyluk J, Perron A, Houde M, Limin A, Fowler B, et al (1998) Accumulation of an acidic dehydrin in the vicinity of the plasma membrane during cold acclimation of wheat. Plant Cell 10: 623–638. 9548987 10.1105/tpc.10.4.623 PMC144014

[22] Houde M, Dallaire S, N'Dong D, Sarhan F (2004) Overexpression of the acidic dehydrin WCOR410 improves freezing tolerance in transgenic strawberry leaves. Plant Biotechnol J 2: 381–387. 17168885 10.1111/j.1467-7652.2004.00082.x

[23] Lin CT, Wei WG, Everson E, Thomashow MF (1990) Cold-acclimation in *Arabidopsis* and wheat: A response associated with expression of related genes encoding ‘boiling-stable’ polypeptides. Plant Physiol 94; 1078–1083. 10.1104/pp.94.3.1078 PMC1077344 16667799

[24] Grossi M, Gulli M, Stanca AM, Cattivelli L (1995) Characterization of two barley genes that respond rapidly to dehydration stress. Plant Sci 105: 71–80.

[25] Lee SC, Lee MY, Kim SJ, Jun SH, An G, et al (2005) Characterization of an abiotic stress-inducible dehydrin gene, *OsDhn1*, in rice (*Oryza sativa* L.). Mol Cells 19: 212–218. 15879704

[26] Choi DW, Zhu B, Close TJ (1999) The barley (*Hordeum vulgare* L.) dehydrin multigene family: sequences, allele types, chromosome assignments, and expression characteristics of 11 *Dhn* genes of cv Dicktoo. Theor Appl Genet 98: 1234–1247.

[27] Welin BV, Olson A, Nylander M, Palva ET (1994) Characterization and differential expression of *dhn/lea/rab*-like genes during cold-acclimation and drought stress in *Arabidopsis thaliana* . Plant Mol Biol 26: 131–144. 7948863 10.1007/BF00039526

[28] James VA, Neibaur I, Altpeter F (2008) Stress inducible expression of the DREB1A transcription factor from xeric, *Hordeum spontaneum* L. in turf and forage grass (*Paspalum notatum* Flugge) enhances abiotic stress tolerance. Transgenic Res 17: 93–104. 17415675 10.1007/s11248-007-9086-y

[29] Morran S, Eini O, Pyvovarenko T, Parent B, Singh R, et al (2011) Improvement of stress tolerance of wheat and barley by modulation of expression of DREB/CBF factors. Plant Biotechnol J 9: 230–249. 20642740 10.1111/j.1467-7652.2010.00547.x

[30] Cenci A, Chantret N, Kong X, Gu Y, Anderson OD, et al (2003) Construction and characterization of a half million clone BAC library of durum wheat (*Triticum turgidum* ssp *durum*). Theor Appl Genet 107: 931–939. 12830387 10.1007/s00122-003-1331-z

[31] Kovalchuk N, Smith J, Pallotta M, Singh R, Ismagul A, et al (2009) Characterization of the wheat endosperm transfer cell-specific protein TaPR60. Plant Mol Biol 71: 81–98. 19513805 10.1007/s11103-009-9510-1

[32] Curtis MD, Grossniklaus U (2003) A gateway cloning vector set for high-throughput functional analysis of genes in planta. Plant Physiol 133: 462–469. 14555774 10.1104/pp.103.027979 PMC523872

[33] Shimada T, Sasakuma T, Tsunewaki K (1969) In vitro culture of wheat tissues. I. Callus formation, organ redifferentiation and single cell culture. Can J Genet Cytol 11: 294–304.

[34] Sanford JC, Smith FD, Russell JA (1993) Optimizing the biolistic process for different biological applications. Meth Enzymol 217: 483–509. 8474348 10.1016/0076-6879(93)17086-k

[35] Li M, Singh R, Bazanova N, Milligan AS, Shirley N, et al (2008) Spatial and temporal expression of endosperm transfer cell-specific promoters in transgenic rice and barley. Plant Biotechnol J 6: 465–476. 18422887 10.1111/j.1467-7652.2008.00333.x

[36] Lopato S, Bazanova N, Morran S, Milligan AS, Shirley N, et al (2006) Isolation of plant transcription factors using a modified yeast one-hybrid system. Plant Methods 2: 3–15. 16504065 10.1186/1746-4811-2-3 PMC1402289

[37] Sali A, Blundell TL (1993) Comparative protein modelling by satisfaction of spatial restraints. J Mol Biol 234: 779–815. 8254673 10.1006/jmbi.1993.1626

[38] Kelley LA, MacCallum RM, Sternberg MJE (2000) Enhanced genome annotation using structural profiles in the program 3D-PSSM. J Mol Biol 299: 499–520. 10860755 10.1006/jmbi.2000.3741

[39] Wu ST, Zhang Y (2007) LOMETS: A local meta-threading-server for protein structure prediction. Nucl Acids Res 35: 3375–3382. 17478507 10.1093/nar/gkm251 PMC1904280

[40] Wu ST, Zhang Y (2008) MUSTER: Improving protein sequence profile-profile alignments by using multiple sources of structure information. Proteins 72: 547–556. 18247410 10.1002/prot.21945 PMC2666101

[41] Ginalski K, Pas J, Wyrwicz LS, von Grotthuss M, Bujnicki JM, et al (2003) ORFeus: detection of distant homology using sequence profiles and predicted secondary structure. Nucl Acids Res 31: 3804–3807. 12824423 10.1093/nar/gkg504 PMC168911

[42] Allen MD, Yamasaki K, Ohme-Takagi M, Tateno M, Suzuki M (1998) A novel mode of DNA recognition by a beta-sheet revealed by the solution structure of the GCC-box binding domain in complex with DNA. EMBO J 17: 5484–5496. 9736626 10.1093/emboj/17.18.5484 PMC1170874

[43] Corpet F, Gouzy J, Kahn D (1998) The ProDom database of protein domain families. Nucl Acids Res 26: 323–326. 9399865 10.1093/nar/26.1.323 PMC147246

[44] Pei JM, Tang M, Grishin NV (2008) PROMALS3D web server for accurate multiple protein sequence and structure alignments. Nucl Acids Res 36: W30–W34. 18503087 10.1093/nar/gkn322 PMC2447800

[45] Callebaut I, Labesse G, Durand P, Poupon A, Canard L, et al (1997) Deciphering protein sequence information through hydrophobic cluster analysis (HCA): current status and perspectives. Cell Mol Life Sci 53: 621–645. 9351466 10.1007/s000180050082 PMC11147222

[46] Laskowski RA, Macarthur MW, Moss DS, Thornton JM (1993) PROCHECK: A program to check the steriochemical quality of protein structures. J Appl Crystallogr 26: 283–291.

[47] Sippl MJ (1993) Recognition of errors in three-dimensional structures of proteins. Proteins 17: 355–362. 8108378 10.1002/prot.340170404

[48] Pei JM, Kim BH, Grishin NV (2008) PROMALS3D: a tool for multiple protein sequence and structure alignments. Nucl Acids Res 36: 2295–2300. 18287115 10.1093/nar/gkn072 PMC2367709

[49] Page RDM (1996) TreeView: An application to display phylogenetic trees on personal computers. Comp Appl Bio Sci 12: 357–358. 10.1093/bioinformatics/12.4.357 8902363

[50] Burton RA, Jobling SA, Harvey AJ, Shirley NJ, Mather DE, et al (2008) The genetics and transcriptional profiles of the cellulose synthase-like *HvCslF* gene family in barley. Plant Physiol 146: 1821–1833. 18258691 10.1104/pp.107.114694 PMC2287353

[51] Yamaguchi-Shinozaki K, Shinozaki K (2005) Organization of *cis*-acting regulatory elements in osmotic- and cold-stress-responsive promoters. Trends Plant Sci 10: 88–94. 15708346 10.1016/j.tplants.2004.12.012

[52] Higo K, Ugawa Y, Iwamoto M, Korenaga T (1999) Plant *cis*-acting regulatory DNA elements (PLACE) database: 1999. Nucl Acids Res 27: 297–300. 9847208 10.1093/nar/27.1.297 PMC148163

[53] Prestridge DS (1991) SIGNAL SCAN: a computer program that scans DNA-sequences for eukaryotic transcriptional elements. Comp Appl Bio Sci 7: 203–206. 10.1093/bioinformatics/7.2.203 2059845

[54] Baker SS, Wilhelm KS, Thomashow MF (1994) The 5'-region of *Arabidopsis thaliana cor15a* has *cis*-acting elements that confer cold-, drought- and ABA-regulated gene expression. Plant Mol Biol 24: 701–713. 8193295 10.1007/BF00029852

[55] Liu Q, Kasuga M, Sakuma Y, Abe H, Miura S, et al (1998) Two transcription factors, DREB1 and DREB2, with an EREBP/AP2 DNA binding domain separate two cellular signal transduction pathways in drought- and low-temperature-responsive gene expression, respectively, in Arabidopsis. Plant Cell 10: 1391–1406. 9707537 10.1105/tpc.10.8.1391 PMC144379

[56] Yamaguchi-Shinozaki K, Urao T, Iwasaki T, Kiyosue T, Shinozaki K (1994) Function and regulation of genes that are induced by dehydration stress in *Arabidopsis thaliana*. JIRCAS J: 69–79.

[57] Cao WH, Liu J, Zhou QY, Cao YR, Zheng SF, et al (2006) Expression of tobacco ethylene receptor NTHK1 alters plant responses to salt stress. Plant Cell Envir 29: 1210–1219. 10.1111/j.1365-3040.2006.01501.x 17080944

[58] Fujimoto SY, Ohta M, Usui A, Shinshi H, Ohme-Takagi M (2000) Arabidopsis ethylene-responsive element binding factors act as transcriptional activators or repressors of GCC box-mediated gene expression. Plant Cell 12: 393–404. 10715325 10.1105/tpc.12.3.393 PMC139839

[59] Ohta M, Matsui K, Hiratsu K, Shinshi H, Ohme-Takagi M (2001) Repression domains of class II ERF transcriptional repressors share an essential motif for active repression. Plant Cel 13: 1959–1968. 10.1105/TPC.010127 PMC139139 11487705

[60] Ohta M, Ohme-Takagi M, Shinshi H (2000) Three ethylene-responsive transcription factors in tobacco with distinct transactivation functions. Plant J 22: 29–38. 10792818 10.1046/j.1365-313x.2000.00709.x

[61] Sasaki K, Mitsuhara I, Seo S, Ito H, Matsui H, Ohashi Y (2007) Two novel AP2/ERF domain proteins interact with *cis*-element VWRE for wound-induced expression of the Tobacco *tpoxN1* gene. Plant J 50: 1079–1092. 17488240 10.1111/j.1365-313X.2007.03111.x

[62] Yang Z, Tian LN, Latoszek-Green M, Brown D, Wu KQ (2005) *Arabidopsis* ERF4 is a transcriptional repressor capable of modulating ethylene and abscisic acid responses. Plant Mol Biol 58: 585–596. 16021341 10.1007/s11103-005-7294-5

[63] Ashkenazy H, Erez E, Martz E, Pupko T, Ben-Tal N (2010) ConSurf 2010: calculating evolutionary conservation in sequence and structure of proteins and nucleic acids. Nucl Acids Res 38: W529–533. 20478830 10.1093/nar/gkq399 PMC2896094

[64] McGuffin LJ, Bryson K, Jones DT (2000) The PSIPRED protein structure prediction server. Bioinformatics 16: 404–405. 10869041 10.1093/bioinformatics/16.4.404

[65] Karplus K (2009) SAM-T08, HMM-based protein structure prediction. Nucl Acids Res 37: W492–W497. 19483096 10.1093/nar/gkp403 PMC2703928

[66] Frishman D, Argos P (1995) Knowledge-based protein secondary structure assignment. Proteins 23: 566–579. 8749853 10.1002/prot.340230412

[67] Kabsch W, Sander C (1983) How good are predictions of protein secondary structure? FEBS Lett 155: 179–182. 6852232 10.1016/0014-5793(82)80597-8

[68] Kim DE, Chivian D, Baker D (2004) Protein structure prediction and analysis using the Robetta server. Nucl Acids Res 32: W526–W531. 15215442 10.1093/nar/gkh468 PMC441606

[69] Lascombe MB, Bakan B, Buhot N, Marion D, Blein JP, et al (2008) The structure of "defective in induced resistance'' protein of *Arabidopsis thaliana*, DIR1, reveals a new type of lipid transfer protein. Prot Sci 17: 1522–1530. 10.1110/ps.035972.108 PMC2525531 18552128

[70] Smith TF, Waterman MS (1981) Identification of common molecular subsequences. Journal of Mol Biol 147: 195–197. 7265238 10.1016/0022-2836(81)90087-5

[71] Sali A, Potterson L, Yuan F, van Vlijmen H, Karplus M (1995) Evaluation of comparative protein modeling by MODELLER. Proteins 23: 318–326. 8710825 10.1002/prot.340230306

[72] Pasquato L, Pengo P, Scrimin P (2005) Nanozymes: Functional nanoparticle-based catalysts. Supramol Chem 17: 163–171.

[73] Thomashow MF (1999) Plant cold acclimation: Freezing tolerance genes and regulatory mechanisms. Ann Rev Plant Physiol Mol Biol 50: 571–599. 10.1146/annurev.arplant.50.1.571 15012220

[74] Buttner M, Singh KB (1997) *Arabidopsis thaliana* ethylene-responsive element binding protein (AtEBP), an ethylene-inducible, GCC box DNA-binding protein interacts with an *ocs* element binding protein. Proc Natl Acad Sci USA 94: 5961–5966. 9159183 10.1073/pnas.94.11.5961 PMC20889

[75] Cao YF, Song FM, Goodman RM, Zheng Z (2006) Molecular characterization of four rice genes encoding ethylene-responsive transcriptional factors and their expressions in response to biotic and abiotic stress. J Plant Physiol 163 1167–1178. 10.1016/j.jplph.2005.11.004 16436304

[76] Chen XJ, Guo ZJ (2008) Tobacco OPBP1 enhances salt tolerance and disease resistance of transgenic rice. Int J Mol Sci, 9, 2601–2613. 10.3390/ijms9122601 PMC2635653 19330095

[77] Hao DY, Yamasaki K, Sarai A, Ohme-Takagi M (2002) Determinants in the sequence specific binding of two plant transcription factors, CBF1 and NtERF2, to the DRE and GCC motifs. Biochemistry 41: 4202–4208. 11914065 10.1021/bi015979v

[78] Huang Z, Huang R, Huang D (2004) ERF transcription factors and their roles in plant defense responses. Acta Phytopathol Sinica 34: 193–198.

[79] Kizis D, Lumbreras V, Pages M (2001) Role of AP2/EREBP transcription factors in gene regulation during abiotic stress. FEBS Lett 498: 187–189. 11412854 10.1016/s0014-5793(01)02460-7

[80] Lin R, Zhao W, Meng X, Peng YL (2007) Molecular cloning and characterization of a rice gene encoding AP2/EREBP-type transcription factor and its expression in response to infection with blast fungus and abiotic stresses. Physiol Mol Plant Pathol 70: 60–68.

[81] Solano R, Ecker JR (1998) Ethylene gas: perception, signaling and response. Curr Opin Plant Biol 1: 393–398. 10066624 10.1016/s1369-5266(98)80262-8

[82] Tournier B, Sanchez-Ballesta MT, Jones B, Pesquet E, Regad F, et al (2003) New members of the tomato ERF family show specific expression pattern and diverse DNA-binding capacity to the GCC box element. FEBS Lett 550: 149–154. 12935902 10.1016/s0014-5793(03)00757-9

[83] Lee JH, Hong JP, Oh SK, Lee S, Choi D, et al (2004) The ethylene-responsive factor like protein 1 (CaERFLP1) of hot pepper (*Capsicum annuum* L.) interacts *in vitro* with both GCC and DRE/CRT sequences with different binding affinities: Possible biological roles of CaERFLP1 in response to pathogen infection and high salinity conditions in transgenic tobacco plants. Plant Mol Biol 55: 61–81. 15604665 10.1007/s11103-004-0417-6

[84] Zhang ZY, Yao WL, Dong N, Liang HX, Liu HX, et al (2007) A novel ERF transcription activator in wheat and its induction kinetics after pathogen and hormone treatments. J Exp Bot 58: 2993–3003. 17728298 10.1093/jxb/erm151

[85] Kagale S, Links MG, Rozwadowski K (2010) Genome-wide analysis of ethylene-responsive element binding factor-associated amphiphilic repression motif-containing transcriptional regulators in Arabidopsis. Plant Physiol 152: 1109–1134. 20097792 10.1104/pp.109.151704 PMC2832246

[86] Ciftci-Yilmaz S, Morsy MR, Song L, Coutu A, Krizek BA, et al (2007) The EAR-motif of the Cys2/His2-type zinc finger protein Zat7 plays a key role in the defense response of *Arabidopsis* to salinity stress. J Biol Chem 282: 9260–9268. 17259181 10.1074/jbc.M611093200

[87] Wang S, Yang S, Yin Y, Xi J, Li S, et al (2009) Molecular dynamics simulations reveal the disparity in specific recognition of GCC-box by AtERFs transcription factors super family in *Arabidopsis* . J Mol Recogn 22: 474–479. 10.1002/jmr.965 19533627

