# Supplementary material for: Complex Regulation by Apetala2 Domain-Containing Transcription Factors Revealed through Analysis of the Stress-Responsive TdCor410b Promoter from Durum Wheat
Source: PLoS One. 2013 Mar 19;8(3):e58713. doi: 10.1371/journal.pone.0058713 (PMC3602543; doi:10.1371/journal.pone.0058713)
Supplement: Table S1 — List of primers used for Q-PCR. (DOCX) [file pone.0058713.s007.docx]

Table S1. List of primers used for Q-PCR.

| **Short gene name** | **Full gene name** | **Forward primer** | **Reverse primer** | **Size of the PCR fragment (bp)** | **Acquisition temperature (^o^C)** |
| --- | --- | --- | --- | --- | --- |
| *HvGAPdH* | *Glyceraldehyde-3-phosphate dehydrogenase* | GTGAGGCTGGTGCTGATTACG | TGGTGCAGCTAGCATTTGAGAC | 198 | 82 |
| *HvCyclophilin* | *Cyclophilin* | CCTGTCGTGTCGTCGGTCTAAA | ACGCAGATCCAGCAGCCTAAAG | 122 | 81 |
| *HvTubulin* | *Alpha tubulin* | AGTGTCCTGTCCACCCACTC | AGCATGAAGTGGATCCTTGG | 248 | 82 |
| *HvHSP70* | *Heat shock protein 70* | CGACCAGGGCAACCGCACCAC | ACGGTGTTGATGGGGTTCATG | 108 | 85 |
| *HvERF4* | *Ethylene responsive factor 4* | GCTTCGGATGCGTCGCAACTC | CCCGTGGATATGATGATGATG | 204 | 85 |
| *TaActin* | *Actin* | GACAATGGAACCGGAATGGTC | GTGTGATGCCAGATTTTCTCCAT | 236 | 80 |
| *TaCyclophilin* | *Cyclophilin* | CAAGCCGCTGCACTACAAGG | AGGGGACGGTGCAGATGAA | 227 | 86 |
| *TaGAPdH* | *Glyceraldehyde-3-phosphate dehydrogenase* | TTCAACATCATTCCAAGCAGCA | CGTAACCCAAAATGCCCTTG | 220 | 80 |
| *TaEFa* | *Elongation factor alpha* | CAGATTGGCAACGGCTACG | CGGACAGCAAAACGACCAAG | 227 | 81 |
| *TaERF4a* | *Ethylene responsive factor 4a* | CGAGAATAGCAAGGATTCGTG | TGCTCGCAGCTTACATGACTG | 229 | 82 |
| *TaERF4b* | *Ethylene responsive factor 4b* | ATAGCAAGGATTCGTGGGAGG | TGTGATCCAGTGAAGCAATGG | 201 | 81 |
| *TaERF5a* | *Ethylene responsive factor 5a* | AGCTGTGATGTACCGCAGGAC | AGGCACCAGACACAAGCACTT | 168 | 79 |
| *TaERF5b* | *Ethylene responsive factor 5b* | AAGATGAAGAAGTGGTGGTGC | GTCTCCAACATGCACCCAATC | 183 | 80 |
| *TaERF6* | *Ethylene responsive factor 6* | GCTAGACAAATGTTAGACCAG | GATCGGGTGGATATTTACAAC | 118 | 80 |
| *TaCor410b* | *Cold responsive protein 410b* | GGACCTCGATTGAATTGTTGG | ATCCAGGACCTTCAAAGACTG | 149 | 81 |
| *TaDREB3* | *Drought responsive element binding protein 3* | CTCGATTCGCTTGCTCCTCAG | TCCTGATGACAAGCTGTAGTGTGC | 163 | 82 |
| *TaDREB2* | *Drought responsive element binding protein 2* | GCGTACAACACCTTGATTTCC | AAACTCAACTCACATCTAAGC | 182 | 76 |
| *TdGAPdH* | *Glyceraldehyde-3-phosphate dehydrogenase* | TTCAACATCATTCCAAGCAGCA | CGTAACCCAAAATGCCCTTG | 220 | 82 |
| *TdEFa* | *Elongation factor alpha* | CAGATTGGCAACGGCTACG | CGGACAGCAAAACGACCAAG | 227 | 80 |
| *TdHSP70* | *Heat shock protein 70* | CGACCAGGGCAACCGCACCAC | ACGGTGTTGATGGGGTTCATG | 108 | 83 |
| *Tdubulin*  *TdERF4a* | *Tubulin*  *Ethylene responsive factor 4a* | AGTGTCCTGTCCACCCACTC  GCTAGACAAATGTTAGACCAG | AGCATGAAGTGGATCCTCGG  GATCGGGTGGATATTTACAAC | 245  118 | 83  80 |
| *TdERF6* | *Ethylene responsive factor 6* | GCTAGACAAATGTTAGACCAG | GATCGGGTGGATATTTACAAC | 118 | 80 |
| *TdCor410b* | *Cold responsive protein 410b* | GGACCTCGATTGAATTGTTGG | ATCCAGGACCTTCAAAGACTG | 149 | 81 |
|  |  |  |  |  |  |
